# Antibody profiling and predictive modeling discriminate between Kaposi sarcoma and asymptomatic KSHV infection

**DOI:** 10.1371/journal.ppat.1012023

**Published:** 2024-02-21

**Authors:** Sydney J. Bennett, Dicle Yalcin, Sara R. Privatt, Owen Ngalamika, Salum J. Lidenge, John T. West, Charles Wood

**Affiliations:** 1 School of Biological Sciences, University of Nebraska-Lincoln, Lincoln, Nebraska, United States of America; 2 Department of Interdisciplinary Oncology, Stanley S. Scott Cancer Center, Louisiana State University Health Sciences Center, New Orleans, Louisiana, United States of America; 3 Dermatology and Venereology Section, University Teaching Hospital, University of Zambia School of Medicine, Lusaka, Zambia; 4 Ocean Road Cancer Institute, Dar es Salaam, Tanzania; 5 Department of Clinical Oncology, Muhimbili University of Health and Allied Sciences, Dar es Salaam, Tanzania; Oregon Health and Science University, UNITED STATES

## Abstract

Protein-level immunodominance patterns against Kaposi sarcoma-associated herpesvirus (KSHV), the aetiologic agent of Kaposi sarcoma (KS), have been revealed from serological probing of whole protein arrays, however, the epitopes that underlie these patterns have not been defined. We recently demonstrated the utility of phage display in high-resolution linear epitope mapping of the KSHV latency-associated nuclear antigen (LANA/ORF73). Here, a VirScan phage immunoprecipitation and sequencing approach, employing a library of 1,988 KSHV proteome-derived peptides, was used to quantify the breadth and magnitude of responses of 59 sub-Saharan African KS patients and 22 KSHV-infected asymptomatic individuals (ASY), and ultimately to support an application of machine-learning-based predictive modeling using the peptide-level responses. Comparing anti-KSHV antibody repertoire revealed that magnitude, not breadth, increased in KS. The most targeted epitopes in both KS and ASY were in the immunodominant proteins, notably, K8.1_29−56_ and ORF65_140-168_, in addition to LANA. Finally, using unbiased machine-learning-based predictive models, reactivity to a subset of 25 discriminative peptides was demonstrated to successfully classify KS patients from asymptomatic individuals. Our study provides the highest resolution mapping of antigenicity across the entire KSHV proteome to date, which is vital to discern mechanisms of viral pathogenesis, to define prognostic biomarkers, and to design effective vaccine and therapeutic strategies. Future studies will investigate the diagnostic, prognostic, and therapeutic potential of the 25 discriminative peptides.

## Author summary

Kaposi sarcoma-associated herpesvirus (KSHV) is the aetiologic agent of sub-Saharan African endemic Kaposi sarcoma (EnKS) and HIV-associated epidemic KS (EpKS). The humoral immune response against KSHV has been studied using infected cell immunofluorescence assays and immunoassays against recombinant proteins. The latency-associated nuclear antigen (LANA) has been established as the most antigenic KSHV protein, and seroreactivity to LANA and the glycoprotein K8.1 are common determinants of KSHV infection. While protein-level antigenicity of a near-complete KSHV proteome has been reported, the epitopes within those proteins have not yet been defined. We have recently demonstrated the utility of phage display in facilitating the first high-resolution epitope mapping of the highly antigenic LANA protein and now apply that same approach to the entire KSHV proteome. Our study presents the most comprehensive and highest-resolution antibody profiling of the KSHV peptidome to date and provides a direct comparison of responses in KS patients and asymptomatic KSHV-seropositive individuals with and without HIV-1 co-infection. The antibody repertoire and epitopes defined here are valuable to furthering the discovery of prognostic biomarkers as well as designing effective preventatives and therapeutics.

## Introduction

Kaposi sarcoma-associated herpesvirus (KSHV), also known as human herpesvirus-8 (HHV-8), is the aetiologic agent for multiple lymphoproliferative disorders, including Kaposi sarcoma (KS), multicentric Castleman’s disease (MCD), primary effusion lymphoma (PEL), and KSHV inflammatory cytokine syndrome (KICS) [1]. KSHV infection is not ubiquitous and seroprevalence varies geographically in accord with the incidence of KS, where co-infection with Human Immunodeficiency Virus 1 (HIV) is predominant [1]. Sub-Saharan Africa (SSA) has the highest prevalence of infections by HIV and KSHV, which correlates with the highest incidence of KS, where the neoplasia is classified into endemic KS (HIV^-^) and epidemic KS (HIV^+^). KS also occurs at high incidence among other high-risk groups: older Mediterranean or Jewish men (classic KS), individuals receiving immunosuppressive treatments (iatrogenic KS), and increasingly, men who have sex with men without HIV infection (HIV^-^ MSM) [1].

KSHV is a gamma-herpesvirus with a linear 165 kbp double-stranded DNA genome surrounded by a capsid, a tegument protein layer, and a lipid envelope. KSHV undergoes a biphasic replication cycle, where during latency, the viral genome exists as an episome tethered to host chromosomes via interaction of its latency-associated nuclear antigen (LANA) with terminal repeats of the viral genome and the host histones 2A/B, without progeny virion production. The latency locus, consisting of ORFK13 (vFLIP), ORF72 (vCyclin), ORF73 (LANA), ORFK12 (Kaposin), and several KSHV encoded micro-RNAs (miRNAs), is expressed during this phase. The resulting latency-associated proteins and miRNAs, along with low-level expression of ORFK1, ORFK15 (LAMP), and ORFK2 (vIL6), promote the survival of the infected cell [1]. KSHV ORF50 encodes the replication and transcription activator (RTA) protein which stimulates the lytic phase of replication upon expression [2]. Lytic reactivation is a staged program of committed expression of the remaining KSHV proteins leading to virion production and cell death. The three stages of lytic reactivation express different groups of genes: *immediate early* genes encode mostly transcription-related genes, *delayed early* genes encode many viral DNA replication-associated proteins, and finally, *late lytic* genes encode mostly viral structural proteins [1].

It was shown that KSHV transmission occurred during childhood in SSA, likely through contact with saliva containing infectious KSHV [3–8]. KSHV PBMC viral load has been associated with the development of KS [9], but plasma viral load was not associated with the response to treatment [10]. The humoral immune response against KSHV has been well investigated, and the presence of anti-KSHV antibody (Ab) is commonly used to diagnose infection. Immunofluorescence assays (IFAs) with PEL cell lines [11–13] and ELISA with recombinant K8.1 and LANA proteins [14,15] have been used to evaluate and quantify protein-level KSHV serological responses. Indeed, detecting LANA-staining in tumor biopsies remains the definitive approach for KS diagnosis. However, seroreversion in both Ab-positive asymptomatic individuals and patients with KSHV-associated disease has been demonstrated [12,16–20]. The presence of neutralizing Ab (nAb) against KSHV has also been studied; we and others have shown that neither total KSHV Ab titer nor the presence of nAb correlated with KSHV viral load [10,14]. We have also found that mothers of KSHV-infected children appeared to have lower total KSHV Ab titers than mothers of children that remained uninfected for two years [16]. Nevertheless, KSHV nAbs were not differential between the two maternal donor groups–a finding that may suggest a role for non-nAbs in the prevention of KSHV transmission [16]. In adults, we have shown that while both KS patients and asymptomatic KSHV-infected individuals (ASY) develop Ab responses, total KSHV Ab titers and KSHV nAb titers were higher in KS patients, suggesting that nAbs are not playing a role once KS developed [11,21]. Whether nAb can play a role in preventing KS development needs further investigation. Moreover, we have previously shown that the prevalence or titer of Ab-dependent cell cytotoxicity (ADCC)-mediating Abs was not differential between KS and ASY [22]. Thus, the role of non-nAbs also needs to be further explored.

Studies on the role of both nAbs and non-nAbs in KSHV disease pathogenesis will require detailed characterization of the antigenicity of the KSHV proteins, including the Ab response against all potential KSHV protein epitopes. Protein-level immunodominance hierarchies against KSHV proteins have demonstrated K8.1/K8.1B, ORF65, ORF38, and ORF73 (LANA) to be the most immunodominant KSHV proteins using protein-based bead arrays [14]. These findings are consistent with immunodominance profiling derived from protein microarrays [23]. The ORF73 C-terminus has also been reported to be more immunodominant than the N-terminus [24]. Expanding on these foundations, in a recent validation of the PhIP-Seq approach, we identified an immunodominant epitope following the LANA central repeat region bridging into the conserved C-terminal domain [25]. Here, we applied a similar strategy to the entire KSHV proteome using a phage library expressing 56-amino acid peptides with 28-amino acid overlap tiled across the viral proteome (VirScan) to identify potential epitopes of all KSHV proteins. We validated the highly antigenic proteins, defined the epitopes within immunodominant proteins, and applied machine learning to build predictive models based on a subset of 25 anti-peptide responses that are discriminative between KS and ASY.

## Results

### Overview of antibody response against the KSHV peptidome

**Fig 1:**
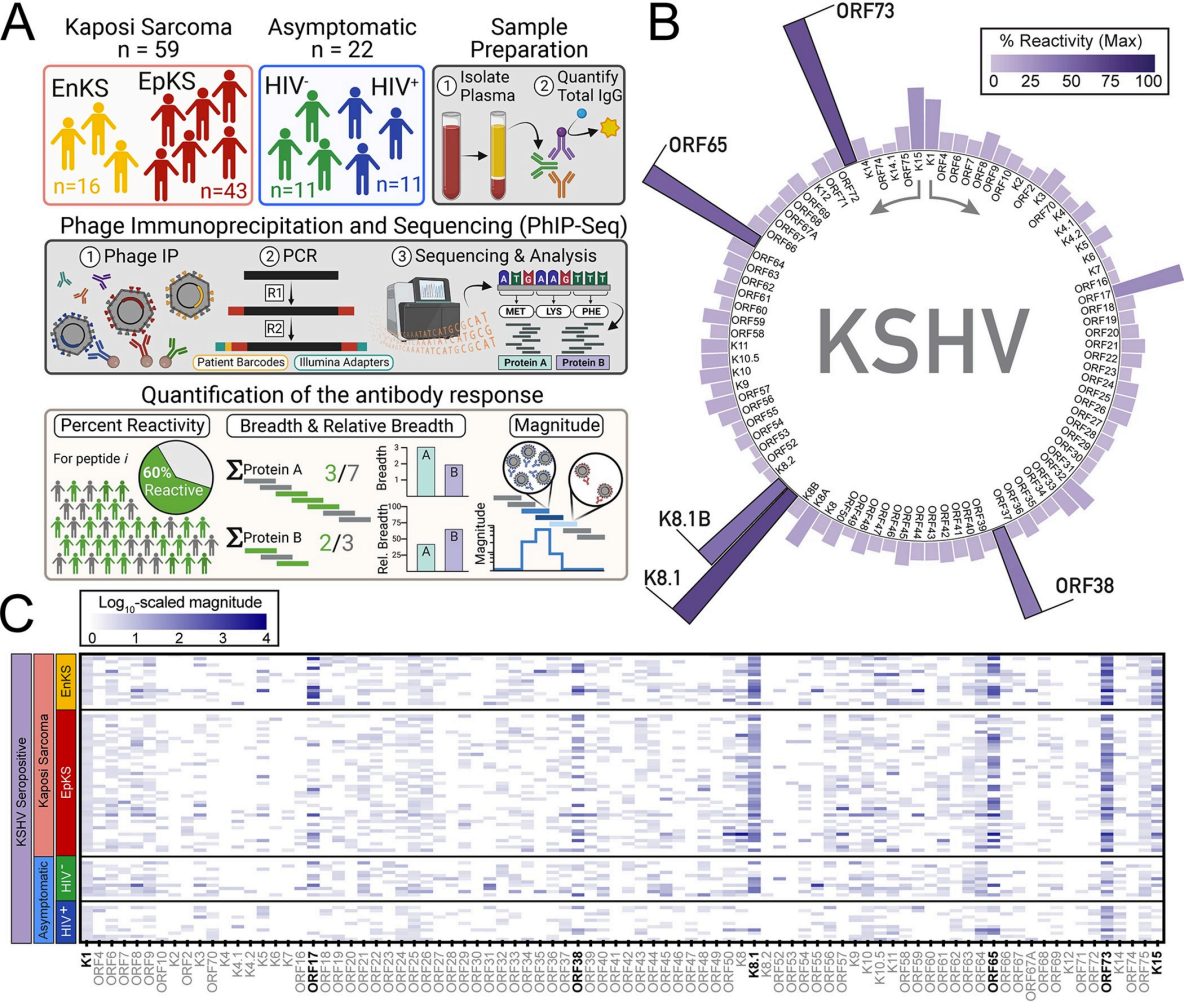
Overview of the antibody responses against the KSHV proteome. **(A)** The antibody repertoire of 59 KS patients and 22 ASY individuals was determined using a phage library expressing 56-mer peptides scanning the entire KSHV proteome. Quantification of the Ab response is represented as breadth (i.e., sum of reactive peptides) and magnitude (i.e., MLXP: -log_10_(*p*)). Created with BioRender.com. **(B)** and **(C)** The log_10_-scaled average magnitude of the antibody responses to KSHV proteins for each individual is represented as a heatmap. See also S1 Fig. Abbreviations: Kaposi Sarcoma (KS), asymptomatic (ASY), phage immunoprecipitation and sequencing (PhIP-Seq), endemic KS (EnKS), epidemic KS (EpKS), human immunodeficiency virus 1 (HIV).

In this study, the VirScan phage library [26] and phage immunoprecipitation and sequencing (PhIP-Seq) [27] were leveraged to comprehensively profile antibody (Ab) responses to 56 amino acid linear/quasi-linear peptides derived from the entire Kaposi Sarcoma-associated herpesvirus (KSHV) (**Fig 1**). The cohort and methods utilized have been previously described [25]. Briefly, Ab-binding repertoires from 59 Kaposi Sarcoma patients (KS) and 22 asymptomatic KSHV-infected individuals (ASY) from sub-Saharan Africa (SSA) were analyzed. We sought to identify (1) antigenicity across the entire KSHV proteome, (2) novel epitopes within immunodominant proteins, and (3) a subset of peptides that could potentially discriminate between KS and ASY (**Figs 1 and S1**). Individuals with and without human immunodeficiency virus 1 (HIV) co-infection were included in both the KS and ASY groups to assess the potential impacts of HIV co-infection on the anti-KSHV repertoire (**Table 1** and **Fig 1A**). Notably, ~50% of the HIV^+^ individuals were aviremic and had normal CD4 counts, allowing evaluation of the effects of HIV suppression on the repertoire against KSHV (**Table 1**).

**Table 1:** Description of the cohort (n = 81). Continuous variables are shown as median with interquartile range (IQR) and compared using Mann-Whitney tests. Categorical data are shown as counts with percentages (%) and compared using Fisher’s Exact tests.

	ASY (n = 22)			KS (n = 59)			
	HIV^-^ ASY	HIV^+^ ASY		EnKS	EpKS		ASY vs. KS
Variable	(n = 11)	(n = 11)	*p*	(n = 16)	(n = 43)	*p*	*p*
Age (years)	33 (5)	43 (12)	*0*.*0781*	54 (33.75)	33 (11)	***0*.*0003***	*0*.*7859*
Sex (male)	5 (45%)	7 (64%)	*0*.*6699*	15 (94%)	26 (63%)	***0*.*0233***	*0*.*2934*
Total IgG (mg/mL)	8.388 (1.63)	8.770 (1.433)	*0*.*7969*	6.279 (5.103)	11.033 (8.822)	***0*.*0250***	*0*.*8289*
KSHV Ab titer	160 (280)	40 (280)	*0*.*4389*	1280 (2240)	1280 (1280)	*0*.*7036*	***<0*.*0001***
> 1:160	5 (45%)	3 (27%)	*0*.*6594*	15 (94%)	37 (86%)	*0*.*6614*	***<0*.*0001***
NGS reads/replicate	1,821,327 (243,137)	1,500,691 (635,943)	***0*.*0440***	1,765,070 (385,470)	1,734,801 (452,445)	*0*.*8815*	*0*.*9634*
HIV duration (days)	---	545 (1615)	*---*	---	180 (670)	*---*	*0*.*1011*
ART (always adhering)	---	11 (100%)	*---*	---	42 (93%)	*---*	*>0*.*9999*
CD4 count	---	360 (191.8)	*---*	---	230.5 (262.5)	*---*	*0*.*0999*
< 200	---	1 (9%)	*---*	---	19 (44%)	*---*	*0*.*1227*
Unknown	---	3 (27%)	*---*	---	1 (2%)	*---*	
HIV viral load (cps/mL)	---	103.5 (43)	*---*	---	2842 (19714.7)	*---*	*0*.*1978*
Undetectable ^a^	---	6 (55%)	*---*	---	18 (42%)	*---*	*0*.*6836*
Unknown	---	3 (27%)	*---*	---	13 (30%)	*---*	

^a^ Undetectable HIV-1 plasma viral load is defined as <70 copies/mL. Abbreviations: asymptomatic KSHV infection (ASY), Kaposi sarcoma (KS), Human Immunodeficiency Virus 1 (HIV), HIV^-^ Endemic KS (EnKS), HIV^+^ Epidemic KS (EpKS), antiretroviral therapy (ART), ***copies*** (cps), antibody (Ab), next-generation sequencing (NGS). This table was modified from a previous publication utilizing this cohort [25].

For each individual, normalized quantities of total IgG (2μg) were added to the VirScan phage library containing the tiled peptide array across the KSHV proteome. After incubation, the Ab-phage complexes were immunoprecipitated using Protein A/G conjugated magnetic beads, the immunoprecipitated phage were lysed, and the phage DNA was PCR amplified (**Fig 1A**, *see Phage IP*). During amplification, each immunoprecipitation was barcoded by individual plasma donors, and Illumina sequencing adapters were added. Then, the DNA libraries were pooled at equimolar levels, gel purified, and sequenced on an Illumina NextSeq 550 by the University of Nebraska Medical Center Genomics Core (**Fig 1A**, *see PCR*). The resulting FASTQ files were aligned to the library oligonucleotides corresponding to all KSHV-derived peptides, and the mapped reads were subjected to pre-processing (**Fig 1A**, *see Sequencing and Analysis*).

The mapped reads were counted, normalized, and fit to a Gamma-Poisson distribution resulting in a *peptide-by-sample* replicate matrix of residual *p*-values (**S1A Fig**). Correlation among replicates was calculated, and only replicates with *Pearson r*_*p*_ *>0*.*7* were included in downstream analyses. Peptides that demonstrated statistically significant enrichment in both replicates (*p<0*.*05*), were deemed *reactive*. Each plate of immunoprecipitations (IPs) contained 8 mock (PBS-only) pull-downs. Peptides that were reactive in >25% of mock IPs were removed from downstream analyses (**S1A Fig**, *see Quality Control*). The final, pre-processed data consisted of the -log_10_(*p*) (MLXPs) of the reactive peptides from concordant replicates with little to no binding in mock IPs. This conservative peptide-level data enabled the quantification of cohort reactivities across the KSHV proteome and identification of epitopes within immunodominant proteins (**S1B Fig**).

Importantly, the phage expressing KSHV peptides were uniformly detected in the input phage library (VirScan) (**S1C Fig**). To better understand the profile of the Ab response to KSHV peptides, we calculated four measures of the Ab response after determining the reactive peptides in each individual: percent reactivity, breadth, relative breadth, and magnitude (**Fig 1A**, *see Quantification of the Ab response*). Percent reactivity represents the proportion of individuals reactive to a peptide (or protein, epitope, etc.). Peaks of percent reactivity were detected across the KSHV peptidome (**S1C Fig**). The percent reactivity against each of the 86 KSHV proteins was summarized by the reactivity to its most targeted constituent peptide across KSHV seropositive individuals (n = 81) (**Fig 1B**); the rationale being that the peptide with the maximal recognition was a logical surrogate quantifier for the relative recognition of the protein as a whole. This approach revealed ORF73 (LANA), K8.1 (glycoprotein), ORF65 (capsid), and ORF38 (tegument) as the most consistently Ab-targeted KSHV proteins. Additionally, the magnitude of the Ab response is defined as the frequency with which a peptide was targeted by Abs and is represented by the MLXP (**Fig 1A**). Thus, for a given peptide, the higher the magnitude, the more Abs in the plasma reacted to that peptide. The magnitude of the Ab response revealed that not only the majority of KSHV seropositive individuals recognized ORF73, K8.1, ORF65, and ORF38, but when recognized, it was with much higher magnitude (**Fig 1C**). These findings reinforce the immunodominance of these proteins, as suggested in previous reports [14,23]. However, while the magnitude of the Ab response reinforced the immunodominance hierarchy, it also revealed substantial heterogeneity between individuals (**Fig 1C**).

### The breadth and magnitude of the antibody response against KSHV

**Fig 2:**
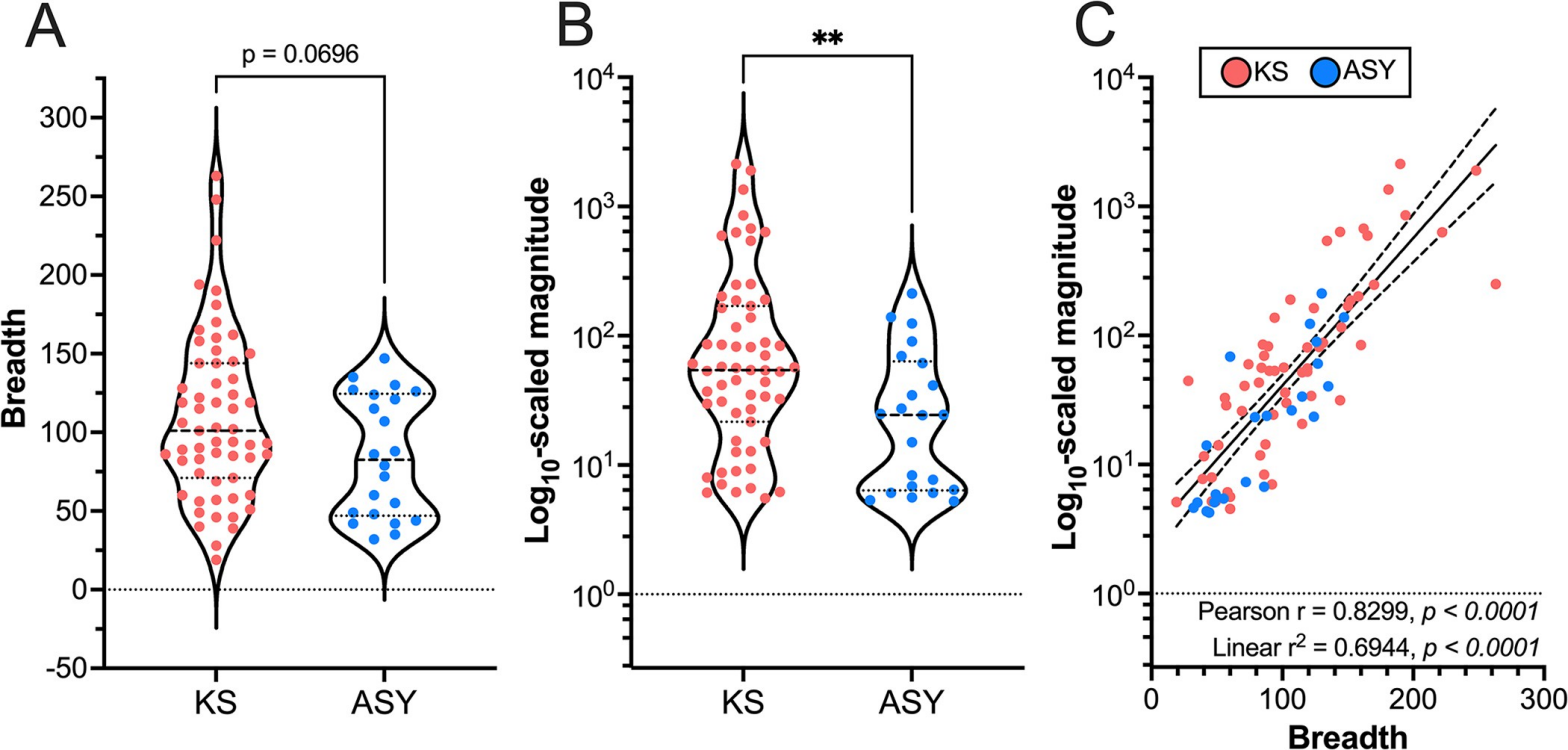
The breadth and magnitude of the antibody response against KSHV. **(A)** The breadth and **(B)** magnitude of the antibody response against KSHV was compared between KS and ASY. Significant comparisons were determined using Mann-Whitney statistical tests. In the violin plots, each data point represents an individual, the dashed lines represent the median, and the dotted lines represent the quartiles. **(C)** Pearson correlation and linear regression of breadth and magnitude, where the solid line represents the goodness-of-ft, and the dashed lines represent the 95% confidence interval. See also S2 and S6 Figs. Abbreviations: Kaposi Sarcoma (KS), asymptomatic (ASY), ****p<0.*0001*, ***p<0.*001*, **p<0.*01*, *p<0.*05*.

Two additional measures describing the KSHV Ab response are breadth and relative breadth. The number of unique peptides to which an individual was reactive is defined as the *breadth*, whereas *relative breadth* is breadth divided by the number of peptides for each protein contained in the library (**Fig 1A**, *see Quantification of the Ab response*). Thus, relative breadth reflects the extent of coverage of Ab reactivity to each protein. While there was a trend toward increased breadth in KS, the number of reactive peptides per KS patient was not significantly different from the number of reactive peptides per ASY individual (**Fig 2A**). In contrast, the average magnitude was significantly increased in KS compared to ASY, meaning that, on average, when a KS patient reacted to a given peptide, they had more Abs targeting that particular peptide (**Fig 2B**). Nevertheless, KSHV breadth and magnitude were highly correlated (**Fig 2C**), indicating that individuals who respond to many KSHV peptides likely respond strongly to those peptides.

Previous reports have shown that KSHV Ab titers were significantly higher in KS than ASY [11]. Plasma anti-KSHV Ab end-point titers were previously determined using an immunofluorescence assay (IFA) against BC3, a chronically infected PEL cell line [25]. Here, we found that both anti-KSHV breadth and magnitude significantly increased with titer; however, they only had a moderate correlation with titer (**S2A–S2D Fig**, *r*_*s*_ *= 0*.*4039 and r*_*s*_ *= 0*.*5391*, respectively). Thus, the titer did not fully explain the breadth and magnitude of the Ab response to KSHV. Detection of higher breadth and magnitude in KS patients was not simply because KS patients tended toward higher titers. To further understand the association between the magnitude of the KSHV Ab response and the KSHV Ab titer, we calculated the average magnitude of response to each KSHV protein and tested for associations with the total KSHV Ab titer. The magnitude of response to a subset of proteins had weak to moderate associations with the KSHV titer, where ORF65, K8.1, K15, and LANA had the strongest associations (**S2E Fig**).

### The antibody response to different functional groups of KSHV proteins

**Fig 3:**
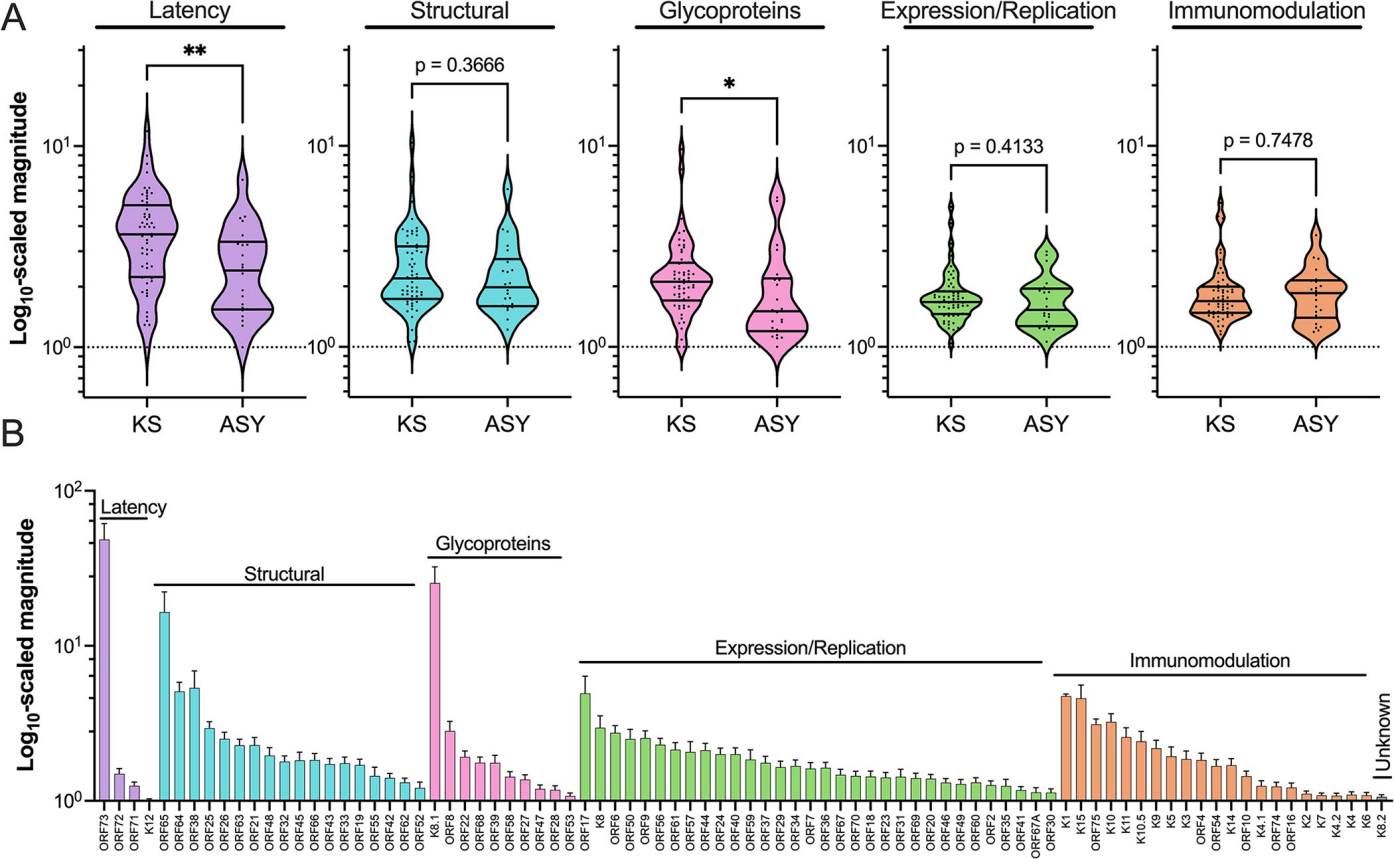
The magnitude of response against functional groups of KSHV proteins. Each KSHV protein was categorized into a functional group, and the magnitude of the antibody response to each functional group as well as the individual protein was compared. Significant comparisons were determined using the Friedman test with Dunn’s multiple comparisons post hoc tests in **(A).** The functional groups were compared between the ASY and KS cohorts. In the volcano plots, each data point represents an individual, the middle line represents the median, and the outer lines represent the quartiles. **(B)** Means with standard errors of the mean (SEM) are shown. See also S3 and S4 Figs. Abbreviations: Kaposi Sarcoma (KS), asymptomatic (ASY), ****p<0.*0001*, ***p<0.*001*, **p<0.*01*, *p<0.*05*.

According to previous literature, each KSHV protein was categorized into one of five functional groups (latency, structural, glycoprotein, expression/replication, immunomodulation) [28] (**Fig 3A**). The structural group consists of capsid and tegument proteins, while the glycoprotein group consists of envelope proteins that would be accessible on the surface membrane. Ab recognition was compared within and between functional groups and between KS/ASY groups. Proteins in the latency, structural, and glycoprotein categories were more targeted than the expression/replication and immunomodulation proteins based on the most frequently recognized proteins in each category (**Fig 3B**). Additionally, we found a significantly increased magnitude of response against latency proteins and glycoproteins in KS compared to ASY, which are likely to be influenced by the responses against ORF73 and K8.1 (**Fig 3A**). Since phage display limits detection to linear epitopes lacking post-translational modifications, it may have missed some Ab responses against glycoproteins. However, we did detect strong responses to K8.1, which is consistent with prior whole protein approaches [14,23] (**Figs 1B–1C** and **3**). Rather than detecting uniform responses to most or all of the proteins in a particular functional group, we found 1–2 immunodominant proteins from particular groups. In addition to the previously described immunogenic proteins (ORF73, K8.1, ORF65, and ORF38), ORF17 (viral protease precursor), K1 (highly variable membrane protein), and K15 (latency-associated membrane protein; LAMP) were the KSHV proteins targeted with the highest magnitude (**Fig 3B**). The relative breadth of the Ab response to the functional groups of KSHV proteins revealed similar trends to the magnitude (**S3 Fig**). These immunodominant proteins were the same proteins whose magnitude was significantly associated with KSHV titer (**S2E Fig**). Finally, the reactivity to the immunodominant proteins more strongly correlated with other immunodominant proteins than with proteins within their functional group (**S4 Fig**). For example, the magnitude of response to ORF73 most correlated with that of K8.1 and K15 rather than the other latency-associated proteins.

### Epitope maps of immunodominant proteins reveal focal points of antigenicity

**Fig 4:**
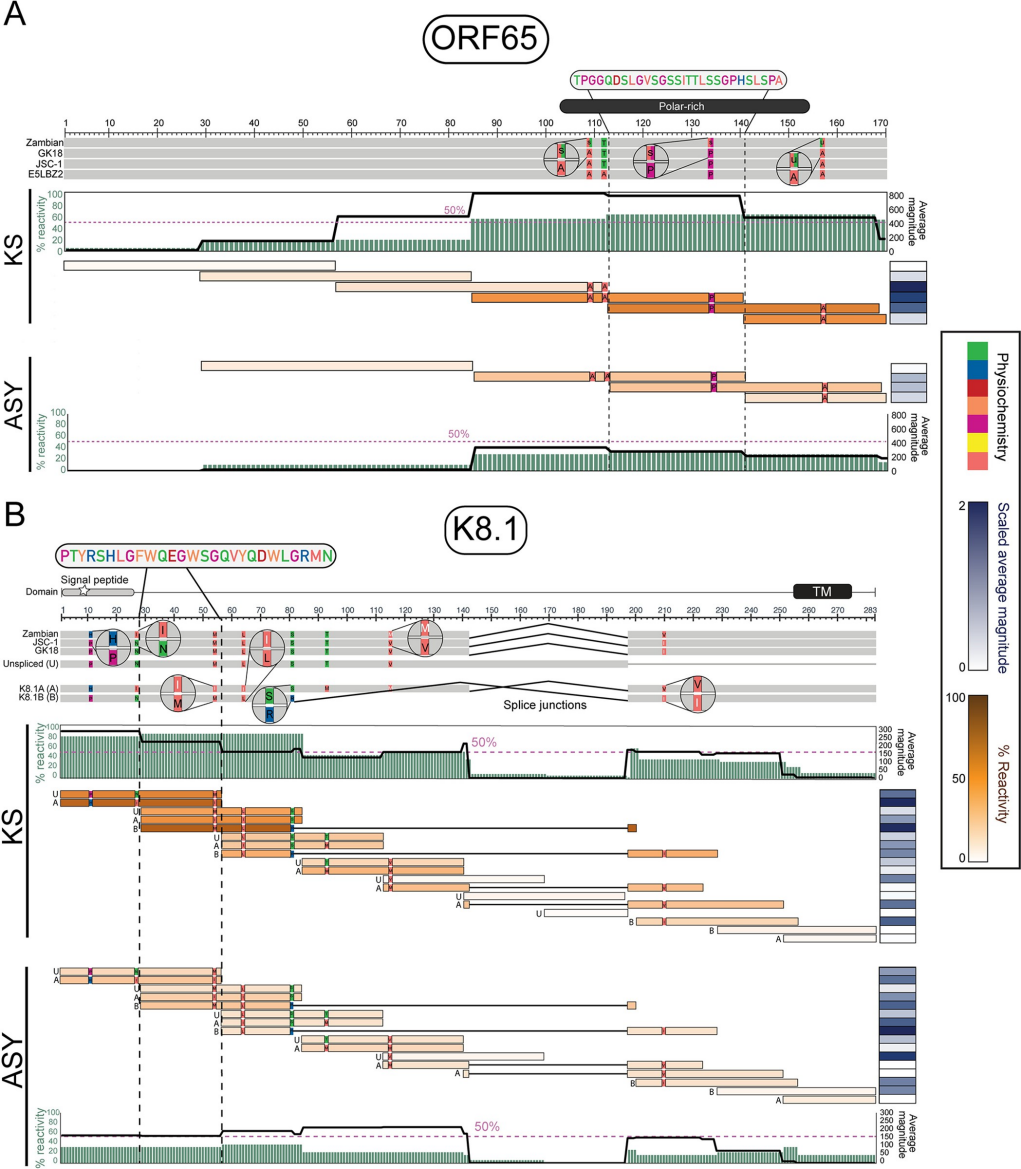
Epitope map of immunodominant proteins–ORF65 (capsid) and K8.1 (glycoprotein). For **(A)** ORF65 and **(B)** K8.1, the known secondary structure and motifs are annotated across the proteins, followed by the multiple sequence alignment (MSA) of reference (GK18, JSC-1) and input library (E5LBZ2, Unspliced, K8.1A, K8.1B) sequences. Gray represents 100% conservation among the sequences, while mismatched residues are colored by their physiochemical properties as defined by the Zappo color scheme (green: hydrophilic, salmon: aliphatic/aromatic, orange: aromatic, fuchsia: conformationally special, yellow: cysteine only, red: negatively charged, blue: positively charged). Further, each peptide targeted by at least one individual was also aligned and colored by the percent reactivity (brown). To the right, the scaled average magnitudes (blue) are shown as a heatmap. Finally, the percent reactivity (sage) and average magnitude (black lines) of the amino acid-level responses are shown for KS and ASY. See also S5 Fig. Abbreviations: Kaposi Sarcoma (KS), asymptomatic (ASY), transmembrane (TM), s: small (A/C/D/G/N/P/S/T/V), u: tiny (A/G/S).

We recently reported the validation of PhIP-seq in epitope mapping of KSHV ORF73 [25]. Here, we extended this approach to define Ab responses against all immunodominant KSHV proteins. ORF65 (small capsid) is essential to viral capsid assembly [29] and was identified as one of the highly immunodominant KSHV proteins in this study and others [14,23]. The percent reactivity and magnitude of the Ab response were mapped across the ORF65 protein at the peptide and amino acid levels (**Fig 4A**). The sequences of the ORF65 peptides included in the phage library were first aligned with a Zambian consensus sequence [30] and two reference sequences (**Fig 4A**, *see top panel*). The Zambian consensus sequence represents the best estimate of the KSHV proteome that is actively circulating in KS and ASY individuals in SSA, while the JSC-1 and GK18 references have been used for genomic and transcriptomic studies [30,31]. Overall, the library peptides were highly similar to the protein antigen to which KS patients in SSA would have likely been infected, as denoted by nearly 100% conservation (gray). Polymorphic residues were highlighted and colored according to their physiochemical properties. Reactivity to ORF65 was not uniform across the protein but instead was focally targeted to the polar-rich region near the C-terminus, ORF65_113-141_ [TPGGQDSLGVSGSSITTLSSGPHSLSPA] (**Fig 4A**, *see middle panel*). This peptide was consistently recognized in KS as indicated by the brown bars (peptide-level) and green columns (residue-level). ORF65_113-141_ was also targeted with high average magnitude in KS as indicated by the dark blue heatmap to the right (peptide-level) and the black line above (residue-level). Albeit with less consistency and lower magnitude, this response was also observed in ASY (**Fig 4A**, *see bottom panel*). Although the peptide immediately preceding ORF65_113-141_ has high average magnitude, only 13% of KS and no ASY were reactive to it, suggesting that it is a private epitope (i.e., patient-specific rather than consistently detected across the cohort).

Epitope mapping of the immunodominant structural protein, ORF38 (tegument) (**S5A Fig**), was confounded by the short length of ORF38–61 amino acids. Since library peptides are 56 amino acids with 28 amino acid overlap, the capacity for finer resolution was limited. Nevertheless, both peptides derived from ORF38 were consistently recognized in KS and ASY, with slightly higher magnitude of detection in KS (**S5A Fig**).

Epitope mapping was also performed for two immunodominant membrane proteins K8.1 and K15 (Type P allele). The glycoprotein K8.1 is often used as part of seroprevalence studies [14], but the epitopes of K8.1 have not previously been mapped. We found that K8.1 was second only to ORF73 (LANA) in percent reactivity. Like ORF65 and ORF38, the K8.1-derived peptides present in the library were found to be representative of the KSHV predicted to circulate in SSA (**Fig 4B**, *see top panel*). The phage library contains K8.1-derived peptides from three splice variants: unspliced, K8.1A, and K8.1B. KS patients responded more consistently to K8.1-derived peptides than ASY (**Fig 4B**, *see middle panel*). A highly antigenic region shared among splice variants was identified immediately following the signal peptide at residues 28–56 [PTYRSHLGFWQEGWSGQVYQDWLGRMN]. ASY individuals responded less consistently, but with similar magnitudes when they did react to K8.1_28−56_ (**Fig 4B**, *see bottom panel*). Importantly, the region unique to the intron in unspliced K8.1 mRNA had little to no reactivity in KS or ASY, as did the transmembrane domain near the C-terminus, reinforcing the specificity of VirScan for identifying appropriate antigenic regions.

Targeting K15 has been suggested to have therapeutic potential [32] and was one of the most antigenic proteins identified in this study, where both high breadth and magnitude of responses were detected (**Figs 1B–1C and 3A**). A focal point of antigenicity was identified in K15 at residues 421–448 [VNRDPPNVFGYASILVSGAEESREPSPQ] in KS patients, but little to no reactivity was evident in ASY (**S5B Fig**). Unfortunately, the antigenic region, K15_421-448_, was predicted to be in the cytoplasmic tail (**S5B Fig**). Thus, while it is interesting that the response to this region is detected in KS and not ASY, therapeutic targeting of this domain is not likely feasible.

### Protein-level Ab responses are differential between KS and ASY

**Fig 5:**
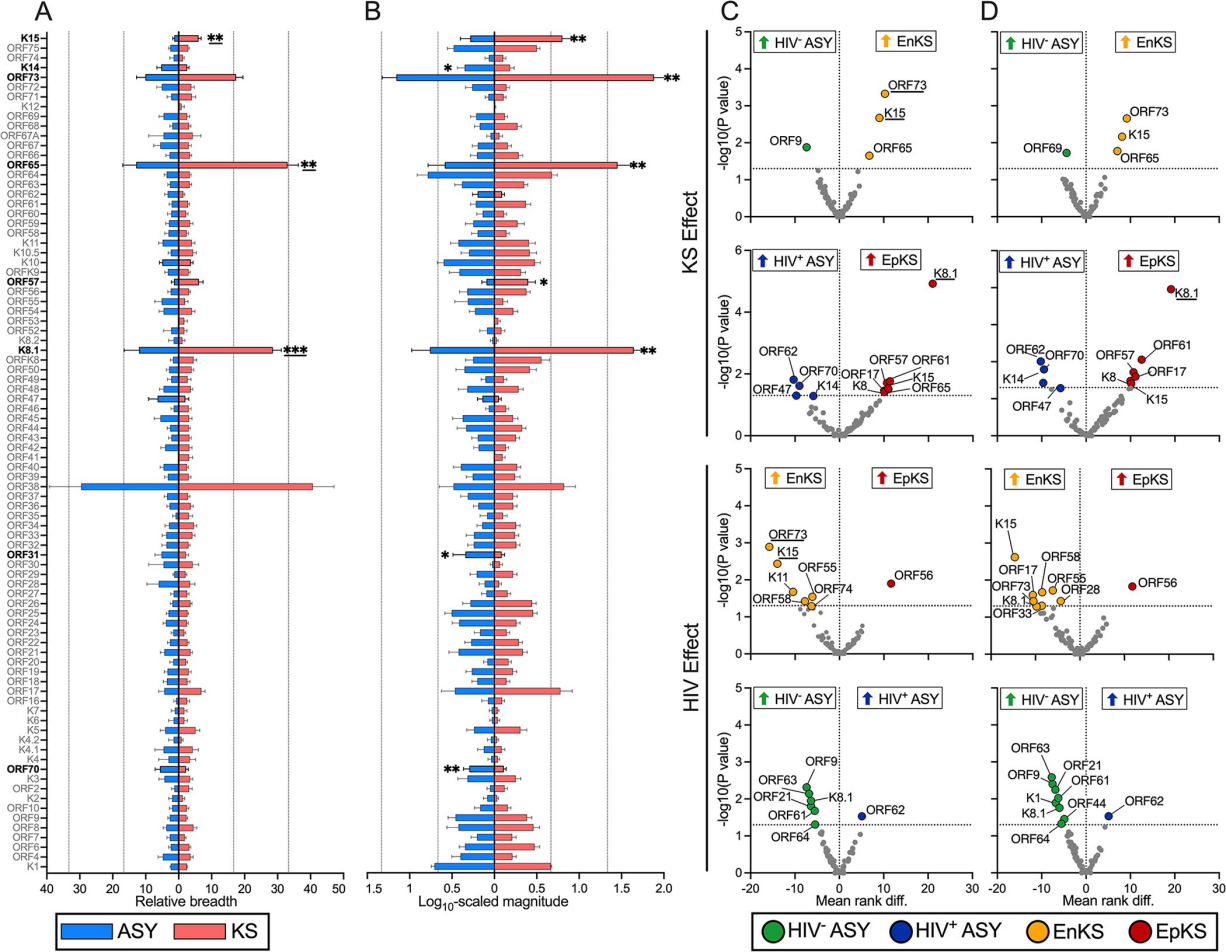
The effect of disease and HIV on the breadth and magnitude of the antibody response to KSHV proteins. **(A)** The breadth and **(B)** magnitude of the antibody response to each KSHV protein were compared between KS and ASY. Means with standard errors of the mean (SEM) are shown. Additionally, the HIV status of the participants was considered when comparing the **(C)** breadth and **(D)** magnitude where each data point in the volcano plot represents a KSHV protein. Significant comparisons were determined using Mann-Whitney tests, and the underlines denote comparisons that remained significant after multiple hypothesis corrections using the two-stage step-up method (Benjamini, Krieger, and Yekutieli; FDR = 10%). See also S6 Fig. Abbreviations: Kaposi Sarcoma (KS), asymptomatic (ASY), human immunodeficiency virus 1 (HIV), endemic KS (EnKS), epidemic KS (EpKS), ****p<0.*0001*, ***p<0.*001*, **p<0.*01*, *p<0.*05*.

Since the immunogenic proteins and epitope mapping did not reveal targets with clear therapeutic or vaccine potential, we asked if the overall breadth and/or magnitude of Ab response could discriminate between KS and ASY and perhaps identify targets with potential diagnostic or prognostic value. Largely, the profiles of the Ab response in ASY mirrored that of KS (**Fig 5A–5B**). There were significant protein-level differences between KS and ASY, and when HIV co-infection was considered, there were many statistically significant differences. However, when we corrected for multiple hypothesis testing (to control the false discovery rate), most of the differences became non-significant at the false discovery rate (FDR) of 10%. However, the number of reactive peptides derived from K8.1, ORF65, and K15 remained statistically significant after multiple hypothesis corrections, indicating that these differences between KS and ASY were unlikely to occur by chance (**Fig 5A**), while none of the protein-level differences in average magnitude remained significant (**Fig 5B**).

To determine the effect of KS alone on the Ab repertoire, KS and ASY were compared after stratification by HIV status. The number of ORF73 and K15 reactive peptides was significantly elevated in EnKS compared to HIV^-^ ASY (**Fig 5C**, *see KS Effect*). In contrast, both the breadth and magnitude of the response against K8.1 were elevated in EpKS compared to HIV^+^ ASY (**Fig 5C–5D**, *see KS Effect*). To evaluate the impact of HIV co-infection, the Ab repertoires of HIV^-^ and HIV^+^ individuals were compared after stratification by KS status. The number of ORF73 and K15 reactive peptides was significantly elevated in EnKS compared to EpKS (**Fig 5C**, *see HIV Effect*), yet Ab responses to KSHV proteins were undifferentiable between HIV^-^ ASY and HIV^+^ ASY (**Fig 5C–5D**, *see HIV Effect*). The significantly differential proteins in these comparisons were the proteins identified as immunodominant by this study and others [14,23].

**Fig 6:**
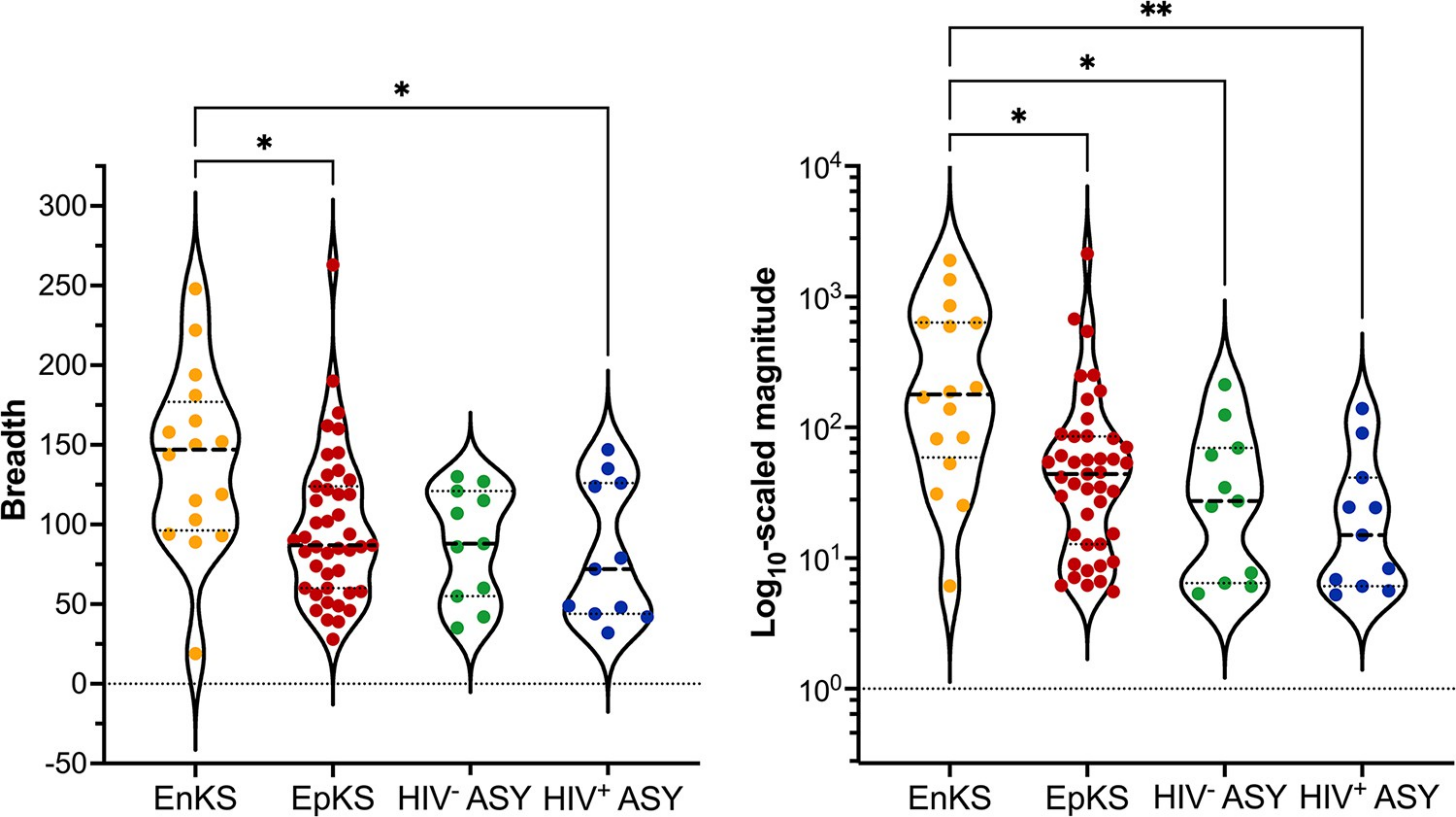
The effect of HIV on the overall antibody response to KSHV in KS and ASY. **(A)** The breadth and **(B)** magnitude of the antibody response to KSHV were compared between EnKS, EpKS, HIV^-^ ASY, and HIV^+^ ASY. Significant comparisons were determined using Kruskal-Wallis with Dunn’s multiple comparisons *post hoc* statistical tests. In the violin plots, each data point represents an individual, the dashed lines represent the median, and the dotted lines represent the quartiles. Abbreviations: Kaposi Sarcoma (KS), asymptomatic (ASY), human immunodeficiency virus 1 (HIV), endemic KS (EnKS), epidemic KS (EpKS), ****p<0.*0001*, ***p<0.*001*, **p<0.*01*, *p<0.*05*.

Overall, EpKS patients had lower breadth and magnitude of Ab response against KSHV than EnKS, while there was no significant difference between HIV^-^ ASY and HIV^+^ ASY (**Fig 6**). Since both HIV and KS affected the breadth and magnitude of the antibody response against the immunodominant KSHV proteins, it was important to determine and compare the effect of HIV co-infection on the percent reactivity and magnitudes at higher resolution of the regions covering the epitopes, in parallel with previous comparisons for anti-ORF73 (LANA) responses [25]. Focusing on the top four immunodominant proteins, our data clearly shows that, regardless of HIV status, most of EpKS patients can respond as consistently to the conserved epitope regions of immunodominant proteins as the EnKS patients (**S6A–S6C Fig**), with K15 as an exception. K15 exhibited substantially more variability in recognition by all samples (**S6D Fig**). In disease, there was a striking decrease in Ab reactivity against K15_421-448_ in EpKS compared to EnKS. However, it is important to note that the EpKS patients who recognized K15 peptides, on average had similarly high magnitudes as EnKS patients. While K8.1 exhibited little to no HIV effect on the percent reactivity at the conserved epitope (**S6C Fig**), there was an average decrease in magnitude among EpKS patients. Lastly, while HIV infection had minimal effects on the antibody response against ORF65 (**S6A Fig**), there was a decrease in both percent reactivity and magnitude against ORF38 in HIV^+^ ASY individuals compared to HIV^-^ ASY. In summary, while overall KSHV breadth was lower in EpKS compared to EnKS, both groups responded consistently to the majority of the immunodominant proteins and their conserved epitopes.

Because Ab reactivity to some of the KSHV proteins significantly differed between KS and ASY, principal component analysis (PCA) was applied to explore whether protein-level responses captured distinct clusters of KS patients and ASY individuals. The protein-level breadth explained 14.5% of the total variance by the first two principal components (**S7A Fig**), whereas the protein-level magnitude explained 29.7% (**S7B Fig**). While the protein-level magnitude of responses separated KS from ASY better than protein-level breadth, both data suggested that there are additional or more complex factors of variation that were not well-captured by two principal components. Peptide-level Ab responses, on the other hand, were more discriminative. Peptide-level breadth explained 30.7% of the variance (**S7C Fig**), while the peptide-level magnitude explained 50.9% (**S7D Fig**). Therefore, because peptide-level Ab responses could better explain the variation between KS and ASY, the peptide-level responses were used to extract features for building predictive models.

### Models predictive of KS/ASY status

Preliminary analyses of Ab repertoires suggested higher potential to discriminate between KS and ASY using peptide-level, as opposed to protein-level responses (**S7 Fig**). PCA revealed that the first two principal components captured higher proportion of total variance when using the entire anti-KSHV peptide repertoire instead of the protein-level breadth of responses, indicating that a more discriminant subset of peptide-level Ab responses might exist that, based on the presence, absence, or magnitude of recognition, would distinguish KS from ASY individuals.

In order to build and test unbiased predictive models based on potentially discriminative peptide-level responses, the dataset comprising the presence or absence of reactive peptides was randomly partitioned into ‘training’, ‘validation’, and ‘test’ datasets, with consideration of KSHV disease and HIV status in all three datasets (**S9A Fig**, *see Data Partitioning*). The test set, comprising 25% of the total observations, serves as unseen data to prevent overfitting and evaluate the generalizability of each predictive model. Univariate feature extraction was applied to the training set, where percent cohort reactivities were used to select statistically significant peptides discriminative of KS and ASY **see Methods**). To assess the trade-off between training and test performance (i.e., validation-test trade-off), an additional 25% of the training set was held out specifically as the validation set (**S9A Fig**). To maximize the extraction of features best representative of the entire cohort without oversampling, and to observe the effect of subsampling on the features extracted, the initial data partitioning scheme was repeated five times. Thus, each partitioning yielded a distinct group of discriminative features extracted from the unique training set and resulting in five separate models for building classifiers (**S9A Fig**, *see Feature Extraction*). Multiple classifiers, types of machine learning algorithms that automatically assign classes to unseen data based on patterns learned from an input training dataset, are widely applied to biological datasets. To minimize the influence of a particular classification algorithm, seven state-of-art machine learning algorithms were employed with various optimization parameters and transformation functions (kernels) where possible. This resulted in 28 overall classifiers for comparing the performance of each training model on the test datasets, as well as the training model used (**S8A Fig**).

**Fig 7:**
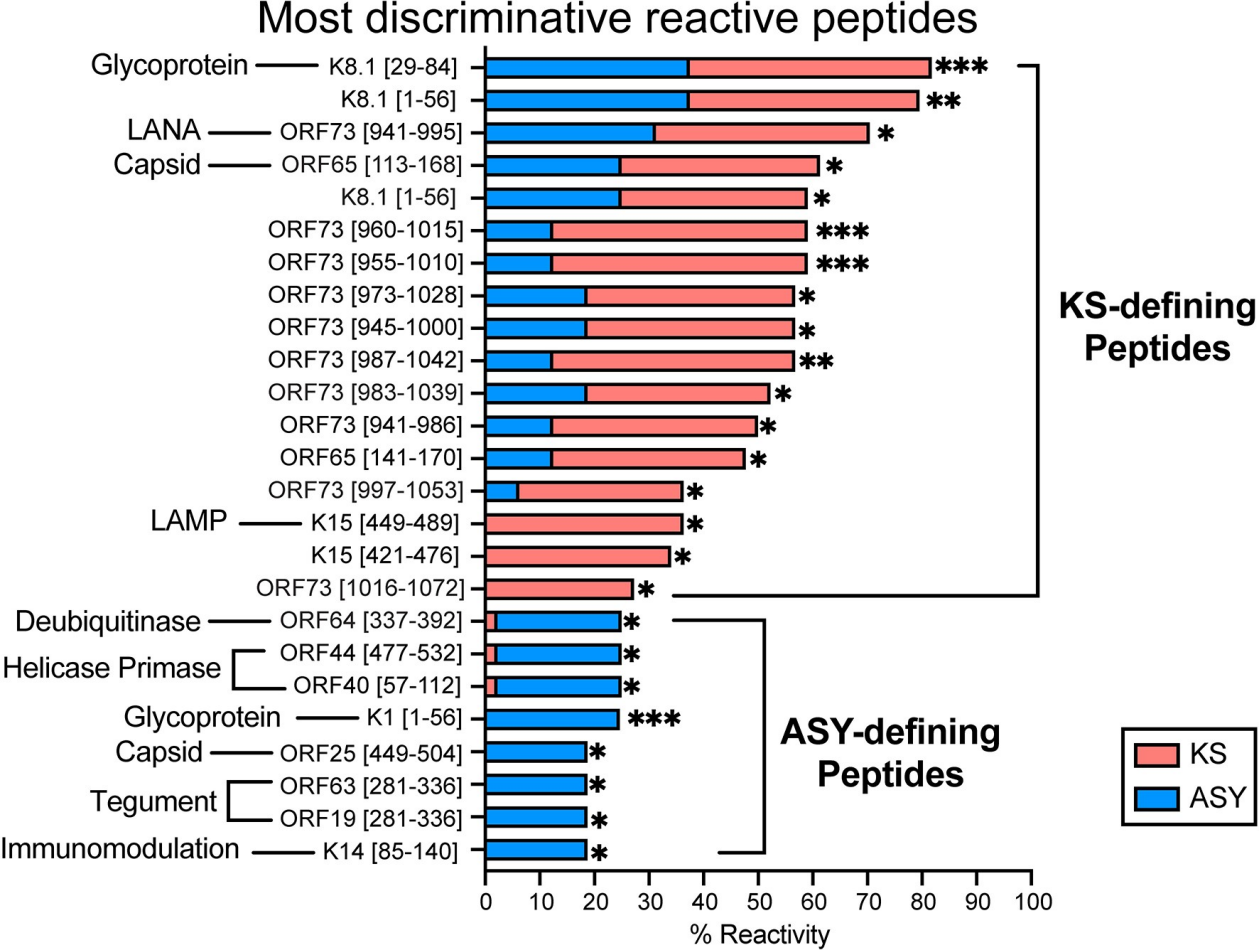
Top discriminative peptides predictive of KS/ASY status. Discriminative peptides from the top-performing model (Model 5) are presented as a superimposed bar plot, where each bar represents the percent reactivity to the given peptide per group. Significant statistical differences in percent reactivities between KS and ASY were determined using Z-test for proportions. See also S7, S8, S9, and S10 Figs. Abbreviations: Kaposi Sarcoma (KS), asymptomatic (ASY), ***p<0.*001*, **p<0.*01*, *p<0.*05*.

The positive predictive value (PPV), negative predictive value (NPV), and area under the receiver operating characteristics (ROC) curve (AUC) were used to objectively assess and compare the performance of each model (**see Methods**). Model 5 demonstrated consistently higher performance on the ‘test’ set, with a median AUC of 0.967 (**S9B Fig**). A majority (16/25) of features in this model were shared with all models; however, the unique combination of 9 additional peptides (K1_1-56_, ORF44_477-532_, ORF19_281-336_, ORF25_449-504_, ORF63_281-336_, ORF64_337-392_, ORF73_1016-1072_, ORF73_997-1053_, and ORF65_113-168_, Fig 7) proved more discriminative in Model 5, implicating those 25 peptides as the top discriminative targets of KSHV Ab responses that predict KS and ASY (**S9B Fig**). Exploratory analyses using PCA supported better linear separability between ASY and KS using only Model 5 features than the total anti-KSHV repertoire (**S8B Fig**).

The top discriminative peptides for both KS- and ASY-defining responses (**Fig 7**) reside in multiple proteins with various functions. Of the KS-predictive peptides, three peptides span the immunodominant epitope of the glycoprotein K8.1, seven peptides span the immunodominant epitope of the LANA C terminus [25], one peptide immediately follows the immunodominant epitope of ORF65, and two peptides encompass and immediately follow the K15 immunodominant epitope (**S10 Fig**). In contrast, the ASY-defining peptides were derived from non-immunodominant KSHV proteins from various functional groups–immunomodulation (K1, K14), virus structure (ORF19, ORF25, ORF63, and ORF64), and expression/replication of viral genes (ORF40, ORF44) (**S10 Fig**). These ASY-defining peptides were reactive in, at most, 20% of ASY, suggesting that combinations of reactivity to these seven peptides define the ASY phenotype. Overall, the reactivity against 25 discriminative peptides was more proficient at discriminating KS and ASY than total KSHV peptide reactivity. The diversity observed in these discriminative features also suggested that ASY-specific Ab responses exist against a set of peptides from various viral proteins, explaining both high PPV and NPV across predictors (**S8A–S8B Fig**).

## Discussion

Elucidation of Ab repertoires is vital to discerning host-pathogen interactions and pathogen-associated disease progression, establishing biomarkers for at-risk individuals or early detection of disease, as well as designing effective vaccine and therapeutic strategies for the prevention of infection or disease progression. In this study, we leveraged the systematic phage peptide display platform VirScan to (1) quantify the antigenicity across the entire KSHV proteome, (2) define novel epitopes within immunodominant proteins, and (3) build predictive models that distinguish KS and ASY.

Our findings were congruent with previous reports [14,23] that ORF73 (LANA), K8.1/K8.1B, ORF65, and ORF38 are the most antigenic KSHV proteins, but we extend those findings to characterize the epitopes of these immunodominant proteins, as well as K15, using similar techniques, as previously described for LANA [25]. The Ab response was not associated with a particular biological function of KSHV proteins (i.e., lytic reactivation); rather, immunodominant peptides derived from the latency (LANA), structural (ORF65/ORF38), and glycoprotein (K8.1) groups. Both HIV co-infection and the development of KS altered the Ab response. The Ab response to KSHV proteins showed variability in the context of HIV co-infection. Expectedly, both breadth and magnitude of KSHV-level responses significantly reduced in HIV co-infected individuals with KS. Since EnKS patients were overall significantly older than EpKS, we investigated whether or not the higher breadth and magnitude observed between EnKS and EpKS was simply due to differences in age. Our analyses revealed no significant correlation between age and the breadth or magnitude of immune response against KSHV (Spearman *ρ* = 0.24, 0.18, respectively). This suggests that the differences observed in immune response between Endemic and Epidemic KS in our cohort are not attributable to age differences. It is also important to note that HIV co-infection had little to no effect on the %-reactivity and average magnitude against the highly targeted epitope within the immunodominant proteins (K8.1, ORF65), however, it did exhibit variability in other immunodominant proteins such as K15 in KS patients and ORF38 in asymptomatic individuals. Finally, consistently targeted epitopes within the immunodominant proteins were identified, and responses to a subset of 25 peptides was found to be discriminative between KS and ASY.

Total anti-KSHV Ab titer has been shown by this study and others [11,25,33,34] to be higher in KS compared to ASY; here, we demonstrated that magnitude, but not breadth, contributed to the titer increase in KS versus ASY. This finding suggests that the KSHV humoral response focuses instead of broadening upon the development of KS. However, we also found a strong correlation between the breadth and magnitude of the KSHV Ab response in both KS and ASY and moderate correlations of titer with overall KSHV breadth and magnitude. Thus, while recall humoral responses to certain epitopes may increase in tumorigenesis, *de novo* responses appear to still develop and expand the breadth of the Ab repertoire in KS patients at a less significant rate than the recall responses. Alternatively, an individual may initially establish breadth in B cell memory during acute infection, and yet not continuously produce Ab at detectable levels from those memory B cells until KS tumorigenesis occurs. The increased lytic expression and cell lysis upon tumorigenesis could result in levels of immunodominant proteins/epitopes that drive anamnestic responses leading to the increase in magnitude with limited expansion of breadth due to the failure to lay down sufficient memory that could persist over the intervening decades with little lytic viral replication before KS development.

Ab repertoires have been previously shown to mark the early detection of virally mediated cancers [35]. The pursuit of discovering prognostic biomarkers for KS pathogenesis in ASY individuals is imperative for the early detection of KS as well as monitoring at risk PLWH, which is met with much greater treatment success than late presentation [36–38]. While protein-level responses were similar between KS and ASY, the peptide-level responses demonstrated better segregation. Using machine-learning-driven predictive models, we found the optimal set of KSHV peptide-level responses that discriminated KS from ASY. For binary classification problems, where the goal is to distinguish between two classes, having features that are characteristic of both categories is vital in building an effective model. The 25 discriminant KSHV peptide responses targeted multiple KSHV proteins and encompassed responses unique to both ASY and KS. Furthermore, positive predictive value (PPV) and negative predictive value (NPV) are important measures of classification performance, especially when features to be validated have potential prognostic value. It is noteworthy that the 25 discriminant peptides resulted not only in high PPV, representative of KS, but also NPV, indicative of ASY. Therefore, the 25 discriminative KSHV peptides defined herein demonstrate a potential for predictive screening to detect early/occult KS pathogenesis in ASY individuals.

Specific Abs against the glycoprotein K8.1 and the gH/gL complex have been shown to have KSHV-neutralizing activity [39,40]. We have previously demonstrated that neutralizing Abs against the gH/gL complex are present in the majority of KSHV seropositive individuals [39]. In addition, K8.1 has long been recognized as an immunodominant protein. Unfortunately, apart from K8.1, we detected only private (i.e., individual-specific) Ab responses to the KSHV glycoproteins (ORF8/gB, ORF22/gH, ORF47/gL, ORF39/gM, ORF53/gN). A whole protein-based bead array system also did not detect strong responses to gB, gH, or gN (gL and gM responses were not measured) [14]. Since nAbs to other viral glycoproteins often target mammalian post-translational modifications or tertiary/quaternary protein structures, VirScan is limited to detecting Ab responses to linear/quasi-linear, continuous epitopes lacking these features. In addition, it is important to note that our study was constrained to plasma-derived Abs, however, the role of mucosal Abs, such as IgA in saliva, in response to KSHV infection would also provide insights into mucosal immunity in natural infection [41]. Future developments and studies should work towards understanding these more complex epitope structures and potential impact of mucosal Ab responses on how they contribute to disease pathogenesis.

Since the more public Abs against the immunodominant epitopes and discriminative peptides identified in this study mainly target intracellular proteins (LANA) or intracellular domains of membrane proteins (K15), they are unlikely to impart neutralizing activity. However, intrabodies–Abs expressed intracellularly–have been considered as therapeutics for infectious diseases and cancers [42,43], and intrabodies specific to KSHV LANA and vIL-6 have demonstrated some pre-clinical therapeutic potential [44–46]. Additionally, non-neutralizing Abs have been shown to provide protection against other viral infections [47], and although we have previously reported that the Ab-dependent cell cytotoxicity (ADCC) activity of plasma-derived Abs was not differential between KS and ASY, ADCC-mediating Abs have been detected in KSHV seropositive individuals, and it is possible that NK cell functional differentials may exist between KS and ASY and could target certain anti-KSHV repertoires better than others [22]. While we were able to identify targets of the KSHV-specific Abs, we did not determine the properties of the KSHV-specific Abs they elicited. Moving forward, it will be essential to understand the properties of these antibodies such as their IgG subtypes, their affinities and avidities as well as their neutralization activities. Whether these responses are differences between the ASY and the KS patients in facilitating better protection in the ASY needs to be determined.

There are some limitations of our study. First, as with any phage display system, our analysis is limited to linear/quasi-linear, continuous epitopes, and given that the phage library was amplified in bacterial cells, the peptides lack mammalian post-translational modifications. Even though conformational or post-translationally modified epitopes will not be present in the phage library, responses to those types of epitopes could be important factors in regulating infection or disease. Although the fact that 56-mer peptides are long enough to adopt some secondary structure, it must be acknowledged that this secondary structure may not represent the folding of the native protein. Nevertheless, based here upon linear peptide recognition, the immunodominant protein hierarchy recapitulated the results of other studies using whole protein approaches [14,23]. Second, immune responses are not only dependent on the presence of an antigen, but also on the inflammatory stimuli, antigen load, role of adaptive immunity, or other KSHV-associated diseases, which might be significant in shaping immunodominance patterns. However, our previous study has shown that the anti-KSHV antibody titers, including nAb titers, and KSHV viral load were similar between EnKS and EpKS, suggesting that chronic inflammation and immune response was not affected by other factors such as immunosuppression and co-infection by HIV. In addition, we have demonstrated no association of KSHV viral load with nAbs [10]. Third, our cohort consisted of exclusively cutaneous KS cases, with no evidence of history of PEL, MCD, or IRIS. Although not directly comparable, in an earlier study by Roshan et al., T-cell responses to KSHV using plasma samples were analyzed [48]. These responses were all weak and lacked immunodominance in KSHV seropositive donors (HIV infected) as well as subjects with multiple KSHV-associated diseases. Therefore, further studies with larger well-designed cohorts are needed to further determine the effects of these co-factors such as disease manifestations, inflammation, prior and/or co-infections, and antigen load on the humoral immune response and the Ab repertoire. Finally, it is important to note that, while the epitopes can be mapped within the overlapping 28-mers, they could still be encompassing multiple epitopes. Future experiments are warranted to resolve these epitopes more precisely.

In summary, this study has provided a deeper understanding of the humoral immune response to KSHV by providing the most comprehensive map of the Ab response to KSHV peptides to date. The epitopes of immunodominant proteins identified in this study will inform future studies on therapeutics and preventatives. Future studies will be needed to experimentally assess and validate the 25 KS- and ASY-defining peptides for prognostic or therapeutic value across larger and longitudinal sample sets within and outside of SSA, and possibly expand the predictive model to include responses to other known human pathogens.

## Materials and methods

### Experimental model and subject details

#### Ethics statement

This study was approved by the Institutional Review Boards of the University of Nebraska, the Louisiana State University Health Sciences Center-New Orleans, the University of Zambia Biomedical Research Ethics Committee, the Tanzania National Institute for Medical Research, and the Ocean Road Cancer Institute. All participants provided written informed consent for the study.

#### Sample collection

Samples were acquired from 16 EnKS, 43 EpKS, 11 HIV^-^ ASY, and 11 HIV^+^ ASY participants from Zambia and Tanzania, as previously described [10,22,25,49–51]. KSHV serology and HIV viral load were previously determined [25] and are presented in Table 1, along with the demographics of the cohort. The EnKS group was significantly older and consisted of more males compared to EpKS, mirroring the disease’s epidemiology [1]. Whole blood samples were collected in EDTA tubes, and plasma was isolated by centrifugation (545x*g* for 15 minutes).

### Method details

#### Library and sample preparation

T7-Vir3 was generously provided by Dr. Stephen J. Elledge at Harvard Medical School [26,52]. Briefly, the library includes 56 amino acid peptides with 28-mer overlap that scan across the KSHV proteome.

The T7-Vir3 library was amplified to ensure that there was enough of the same lot to be used for all plates involved in this study. Amplification and determination of titer were performed as directed by the manufacturer using BLT5403 cells (Novagen T7Select System). We used beads that pulled all IgG subclasses. Total IgG in the plasma was quantified using ELISA (Invitrogen, BMS2091) following the manufacturer’s guidelines, and the plasma was diluted to 0.5μg/μL total IgG.

#### Phage-Immunoprecipitation and Sequencing (PhIP-Seq)

The PhIP-Seq protocol has been reported previously by us and others [25–27,52]. Importantly, each plasma sample was run in duplicate, and 8 mock precipitations (mock IPs), where phosphate-buffered saline (PBS) was added rather than plasma, were run on each plate.

*Complex Formation*. Deep 96-well plates were blocked with 3% BSA in TBST (Tris-buffered saline, 0.1% Tween-20). A single-use aliquot of the amplified T7-Vir3 library was thawed at 4°C and diluted to 2x10^10^ pfu/mL, ensuring 10^5^ pfu per library member for each immunoprecipitation. After pipetting 1mL/well of the prepared library to the blocked deep well plate, 2μg IgG (4μL of 0.5μg/μL plasma dilution) or 4μL PBS was added to the appropriate wells. The phage-plasma mixture was incubated rocking end-over-end at 4°C for 20 hours.

*Immunoprecipitation*. Protein A and Protein G Dynabeads (Invitrogen, 10008D/9D) were mixed 1:1 and 40μL was distributed to each well. The phage-plasma-bead mixture was incubated rocking end-over-end at 4°C for 4 hours. Then, the plate was allowed to sit on a magnetic separation rack (NEB, S1511S) for 2 minutes to pellet the beads. The supernatant was carefully removed with gel-loading tips, and the remaining phage-Ab-bead mixture was resuspended in 400μL PhIP-Seq Wash Buffer (150mM NaCl, 50mM Tris-HCl, and 0.1% (vol/vol) Triton X-100, pH 7.5). After the second wash, the phage-Ab-bead mixture was transferred to a newly blocked 96-well deep well plate. After the third and final wash, the phage-Ab-bead mixture was resuspended in 40μL nuclease-free water and transferred to a 200μL PCR plate. The phage-Ab-bead mixture was placed in a thermocycler at 95°C for 10 minutes before storage at -20°C.

*DNA Library Preparation*. Three rounds of PCR were used to prepare the phage DNA for sequencing using Q5 Hot Start High-Fidelity DNA Polymerase (NEB, M0493L). First, 30 cycles of amplification were performed. Second, eight amplification cycles with primers incorporating the Illumina adaptors and sample barcodes. After adding barcodes and adaptors, the DNA was quantified using qPCR with a TaqMan probe. The barcoded DNA was then pooled into a single tube at equimolar levels. A third round of PCR utilizing a single amplification cycle with replenished primers was performed to ensure the DNA amplicons were of uniform length. Finally, the E.Z.N.A. Gel Extraction Kit (Omega Bio-tek, D2500-02) was used to purify the third-round product (≈376bp).

*DNA Sequencing*. The UNMC Genomics core performed a quality check and sequencing on the gel-purified DNA. DNA concentration was determined using Qubit DS DNA HS Assay reagents and a Qubit Fluorometer (Life Technologies), and size was confirmed using an Agilent 2100 Bioanalyzer. After dilution to 0.3μM with sequencing buffer (following the Illumina custom primer guide), the custom Read1 and Index1 primers were transferred to the reagent cartridge. The gel-purified DNA was denatured with 0.2N NaOH and adjusted to 1.4pM. Clustering and sequencing were performed on an Illumina NextSeq550 using the 50-cycle, single-end protocol (Mid-output flow cell). Illumina Sequence Analysis Viewer was used to monitor the run, and after de-multiplexing, the final FASTQ files were produced.

### Quantification and statistical analysis

#### Phage library annotation

The phage library generated by the VirScan approach contains detailed information about each peptide, which encompasses strain- to kingdom-level taxonomic memberships, amino acid sequence, protein names, and associated UniProt identifiers for the most up-to-date metadata. A unique ID is assigned to each peptide based on the oligonucleotide sequence, which allows for identifying peptide-level duplicate entries. To ensure reliable downstream analyses, an initial round of curations was applied where peptides with obsolete, redundant, or deleted UniProt IDs, as well as duplicate rows, were either removed or had their accession IDs updated based on the UniProtKB. Additionally, to ensure that the quantified Ab-phage complexes represented unique interactions, peptides with identical amino acid sequences were consolidated such that raw read counts obtained from such peptides were summed.

#### Analysis of PhIP-Seq data

*Data Pre-processing*. Initially, reference sequence files were generated in FASTA format, which contained unique oligonucleotides. Based on these, bowtie [53] indices were generated to align the reference sequences to each raw read generated by PhIP-seq. The PhIP-stat repository (https://github.com/lasesonlab/phip-stat) was utilized to align and pre-process the data in multiple steps. First, the sequence alignment generated raw read counts that were derived from each peptide in each replicate. These data were then consolidated, generating a count matrix of peptides vs replicates. A Gamma-Poisson distribution was used to model the variability in the count data, such that, the level of enrichment of each peptide could be estimated under a probabilistic framework. The raw counts were normalized before this step using size factors such that replicates that may have been sequenced to varying depths could be comparable before fitting the model. MLXPs, or -log_10_(*residual p-values*), were used to represent the *magnitude* of Ab responses, reflecting the frequency with which a peptide was targeted. A significance cutoff of 0.05 was used to determine whether a peptide is *reactive*, i.e., an Ab response was confidently detected for the particular peptide, and a presence-absence matrix of the collection of reactive peptides was generated. The sum of all reactive peptides for an individual represented the *breadth* of the Ab response. Breadth can be defined on a peptide-, protein-, or proteome-level, depending on the analysis.

*Quality Control*. Additional to the significance cutoff for determining reactive peptides, we applied a peptide quality cutoff criterion to eliminate non-specific (low-quality) peptides based on their detection in mock IPs. If spurious non-specific binding was observed in more than 25% of the mock IPs for a given peptide, it was filtered out from any downstream analyses. Lastly, Pearson correlation coefficients (*r*_*p*_) were calculated for each replicate pair to determine discordant samples. Samples with r_p_ <0.7 were excluded. Lastly, only the peptides enriched in both replicates were considered reactive for a given individual. Together, our data included only concordant samples and reproducible peptides for quantification of Ab responses.

### Statistical analysis

Briefly, non-parametric statistical tests were used to compare breadth and magnitude among 2 groups (Mann-Whitney) or 3+ groups (Kruskal-Wallis with Dunn’s *post hoc* test for multiple comparisons). Where appropriate, multiple hypothesis correction was applied (two-stage step-up method; Benjamini, Krieger, and Yekutieli; FDR = 10%). Pearson correlations were used to measure the association between linear or normally distributed data, while Spearman correlation coefficients were calculated for assessing associations between non-linear or non-parametric data. Statistical significance was determined if *p<0*.*05*. These statistical analyses were performed in GraphPad Prism v9.3.1.

### Epitope mapping

We obtained full-length sequences of each KSHV protein and its variants present in the VirScan library using the Protein Knowledgebase (UniProtKB). For the specific proteins of which we presented the epitope maps, we obtained the sequences using two reference KSHV proteomes, as well as a Zambian patient-derived KSHV consensus sequence, described in our previous study in detail [25]. To generate epitope maps for each immunodominant protein, the constraint-based multiple alignment tool (COBALT) was utilized with advanced parameters: (1) high introduction, extension, and end gap penalties (-500), (2) using reverse position-specific (RPS)-BLAST (reverse PSI-BLAST) to guide alignments (except ORF65), (3) finding conserved columns and recomputing alignment (except ORF65), and (4) without using query clusters. Alignment mismatches of the primary sequences are highlighted on MSAs and color-coded using the Zappo color scheme, as previously described in detail [25]. The resulting alignment of peptide, library-contained, and reference sequences were exported in scalable vector graphics (SVG) format for downstream data visualization and annotations, including empirically determined functional domains, splicing events, and relevant sequence motifs, as well as the percent reactivity and average magnitude of Ab responses presented for KS and ASY at both peptide- and residue-level.

### Predictive modeling using machine learning

*Training*, *Validation*, *and Test Datasets*. To evaluate the generalizability of the predictive models, 25% of the peptide-level Ab reactivity dataset was used as an unseen (test) dataset (4 EnKS, 11 EpKS, 3 ASY^-^, and 3 ASY^+^), while the remaining 75% of the dataset was used as the training set (12 EnKS, 32 EpKS, 8 ASY^-^, and 8 ASY^+^). Of this training set, 25% hold-out validation was performed for training model assessment. The dataset was partitioned so that the HIV-stratified KS and ASY group sizes were preserved. We randomly resampled five such training, validation, and test datasets without replacement.

*Feature Selection and Classification*. In each of the five resampled datasets for building predictive models, we used the training dataset to calculate percent cohort reactivities. A two-proportions z-test with continuity correction was applied to extract differentially reactive peptides between KS and ASY using the *prop*.*test* function in R. Features with *p<0*.*05* were selected as a feature set for each training dataset. For building unbiased predictive models that predict KS and ASY given selected feature sets, we used a total of 28 classifiers that are grouped into 7 main prediction algorithms widely applied to biological datasets: support vector machine (SVM), linear discriminant analysis (LDA), artificial neural network (ANN), Naïve Bayes classifier (NB), Logistic regression (LR), k-nearest neighbor (KNN), and decision trees (DT). The implementation of all predictive models and their performance assessments were achieved using the Machine Learning Toolbox software package of MATLAB.

*Performance Measures*. As performance measures for evaluating each classifier, we used the area under curve (AUC) of the receiver operating characteristic (ROC) curve. AUC is a metric ranging from 0 to 1 that summarizes the overall performance of the model, where 0.5 indicates a model that performs no better than random guessing and 1 indicates a perfect classifier. The ROC curve plots the false positive rate (FPR, or 1-specificity) against the true positive rate (TPR) at different classification thresholds, providing an objective assessment of the discriminative power of the model, Additionally, we used the positive and negative predictive values (PPV and NPV, respectively), which are statistical measures used to assess the probability that a positive (or negative) result truly indicates the presence (or absence) of a disease in a population. PPV is the proportion of true positives (TP), i.e., individuals who have KS and were predicted as KS, out of all the individuals who are predicted as KS, and is defined as PPV = TP/(TP+FP), where FP represents the false positive cases. A high PPV indicates that there is a high probability that an individual predicted as KS truly has KS in the population being tested. Conversely, a low PPV indicates that an individual predicted as KS is more likely to be a false positive than a true positive. Following a similar logic, for the ASY group, the NPV is defined as NPV = TN/(TN+FN), where TN and FN represent true and false negative, respectively, such that a high NPV indicates that individuals predicted to be ASY are truly ASY.

## Supporting information

S1 Data The relevant data used for analysis throughout this study. Relative breadth and average magnitudes of each KSHV protein per sample are provided, along with group averages. Extensive annotations for the KSHV proteome, including but not limited to genomic order, replication program, functional descriptions are all provided under the “Categories” section. Samples are ordered based on their groups (i.e., EnKS, EpKS, ASY-, ASY+) and color-coded in accordance with the manuscript. A value of “0” indicates no-reactivity.
(XLSX)

S1 Fig Analysis overview with peptide level responses. **(A)** The raw sequencing data was aligned to the reference oligonucleotides, and the mapped reads were counted to obtain a matrix of sample replicates by peptides. Further, raw peptide counts were normalized, and Gamma-Poisson fitted. After residual *p*-values were estimated, the quality of the peptides and samples was assessed. Peptides were excluded if high binding was detected in the mock IPs. Samples were excluded if the replicate correlation coefficient was less than 0.7. Created with BioRender.com. **(B)** The pre-processed peptide-level data was further analyzed at the protein- and epitope-levels. **(C)** The distribution of the normalized counts for each KSHV peptide in the library (black) and the percent reactivity against each peptide for KS (pink) and ASY (blue) is sorted by KSHV genomic order. Abbreviations: Generalized linear model (GLM), Kaposi Sarcoma (KS), asymptomatic (ASY).
(TIF)

S2 Fig Association of titer with breadth and magnitude of protein-level responses. **(A)** The number of reactive peptides from KSHV proteins (Breadth) and **(C)** the frequency with which those peptides were targeted (Magnitude) were compared between individuals with low (*1*:*40*, *1*:*80*, *1*:*160*), medium (*1*:*320*, *1*:*640*, *1*:*1280*), and high (*1*:*2560*, *1*:*5120*, *1*:*10240*) titers. Significant comparisons were determined using Kruskal-Wallis with Dunn’s multiple comparisons *post hoc* test. Each data point represents an individual, and medians with interquartile ranges (IQR) are shown. The Spearman correlation and linear regression of KSHV **(B)** breadth and **(D)** magnitude with titer where the solid lines represent the goodness-of-fit and the dashed lines represent the 95% confidence intervals. **(E)** The average magnitude for each KSHV protein was tested for associations with titer (Spearman). The y-axis represents the correlation coefficient while the color of the point represents the statistical significance. Abbreviations: Kaposi Sarcoma (KS), asymptomatic (ASY), no statistical significance (ns), ****p<0.*0001*, ***p<0.*001*, **p<0.*01*, *p<0.*05*.
(TIF)

S3 Fig The relative breadth of response against functional groups of KSHV proteins. Each KSHV protein was categorized into a functional group, and the breadth of the antibody response to each functional group as well as the individual protein was compared. Significant comparisons were determined using the Friedman test with Dunn’s multiple comparisons *post hoc* tests in **(A)**. The functional groups were compared between the ASY and KS cohorts. In the volcano plots, each data point represents an individual, the middle line represents the median, and the outer lines represent the quartiles. **(B)** Means with standard errors of the mean (SEM) are shown. Kaposi Sarcoma (KS), asymptomatic (ASY), ****p<0.*0001*, ***p<0.*001*, **p<0.*01*, *p<0.*05*.
(TIF)

S4 Fig The correlation of the antibody response against KSHV proteins. The Spearman correlation of log_10_-scaled magnitude and breadth for each KSHV protein with the other KSHV proteins. The proteins that only had weak to no correlation with other KSHV proteins (*r*_*s*_ *< 0*.*4*) are not shown. Abbreviations: open reading frame (ORF).
(TIF)

S5 Fig Epitope maps of additional immunodominant proteins–ORF38 (tegument) and K15 (LAMP). For **(A)** ORF38 and **(B)** K15, the known domains and motifs are annotated across the proteins, followed by the multiple sequence alignment (MSA) of reference (GK18, JSC-1) and input library (CEP3_HHV8P, Q91GT3) sequences. Gray represents 100% conservation among the sequences, while mismatched residues are colored by their physiochemical properties as defined by the Zappo color scheme (green: hydrophilic, salmon: aliphatic/aromatic, orange: aromatic, fuchsia: conformationally special, yellow: cysteine only, red: negatively charged, blue: positively charged). Further, each peptide targeted by at least one individual was also aligned and colored by the percent reactivity (brown). To the right, the scaled average magnitudes (blue) are shown as a heatmap. Finally, the percent reactivity (sage) and average magnitude (black lines) of the amino acid-level responses are shown for KS and ASY. Abbreviations: Kaposi Sarcoma (KS), asymptomatic (ASY), transmembrane (TM), Src homology 2/3 binding sites (SH2/3B), s: small (A/C/D/G/N/P/S/T/V), h: hydrophobic (A/C/F/G/H/I/K/L/M/R/T/V/W/Y), o: alcohol (S/T), t: turn-like (A/C/D/E/G/H/K/N/Q/R/S/T), ∎: resulted in a gap in 5/16 African samples.
(TIF)

S6 Fig The effect of HIV on the antibody response across the immunodominant proteins. The colored areas represent the percentage of patients that were reactive to at least one peptide containing that residue (percent reactivity), while the black lines represent the log_10_-scaled average magnitude [log(-log(*p*))] at that residue for EpKS, EnKS, HIV^+^ ASY, and HIV^-^ ASY in **(A)** ORF65, **(B)** ORF38, **(C)** K8.1, and **(D)** K15. The gray boxes represent the areas of the highlighted epitopes from S4 and S5 Figs. Abbreviations: Kaposi Sarcoma (KS), asymptomatic (ASY), human immunodeficiency virus 1 (HIV), endemic KS (EnKS), epidemic KS (EpKS).
(TIF)

S7 Fig Explorative analysis of KS and ASY antibody responses. 2D principal component analysis (PCA) projections are visualized using the data comprising **(A)** breadth per KSHV protein, **(B)** log_10_-scaled average magnitude per KSHV protein, **(C)** reactive KSHV peptides, and **(D)** log_10_-scaled magnitude for each of the reactive KSHV peptides. In the PCA projections, each data point represents an individual. Abbreviations: Kaposi Sarcoma (KS), asymptomatic (ASY), human immunodeficiency virus 1 (HIV), endemic KS (EnKS), epidemic KS (EpKS), principal component (PC).
(TIF)

S8 Fig Additional Model details. **(A)** Areas under the curve of each tested model are displayed across each classifier for assessing consensus performance between models. The bottom panel shows the positive and negative predictive values using validation and test sets per classifier in the top-performing model (i.e., Model 5). **(B)** 2D PCA projections using all 1,988 KSHV peptides as the feature set (left), compared with the 2D PCA projections using only the 25 discriminative features from the top-performing model–Model 5 (right). Abbreviations: Kaposi Sarcoma (KS), asymptomatic (ASY), area under the curve (AUC), positive predictive value (PPV), negative predictive value (NPV), support vector machine (SVM), k-nearest neighbor (KNN), principal component analysis (PCA).
(TIF)

S9 Fig Additional details on data partitioning, cross validation, and performance comparisons of each model. **(A)** The samples were divided into training (75%) and test sets (25%), preserving sub-class balance within KS and ASY. Five such datasets were generated, using combinations of samples without replacement. Training sets of each dataset were used for unbiased feature extraction. Classification models were built using those extracted features and tested on each partitioned test set. Holdout validation using 25% of the training set was applied to each training set to assess validation-test tradeoffs. The performance of each model was evaluated using positive and negative predictive values (PPV and NPV, respectively), and area under the receiver operating characteristics (ROC) curve (AUC). **(B)** AUCs for each classifier performance within a model were visualized using violin plots, where each point represents the AUC of a classifier and the black line and text represent the median AUC for each model (left). Number of unique or shared peptides in each model are indicated, where Model 5’s features were selected as the top discriminative peptides. Abbreviations: Kaposi Sarcoma (KS), asymptomatic (ASY), human immunodeficiency virus 1 (HIV), endemic KS (EnKS), epidemic KS (EpKS), true negative (TN), true positive (TP), false positive (FP), false negative (FN).
(TIF)

S10 Fig 25 KS and ASY-discriminating peptide sequences derived from each KSHV protein. Multiple peptides spanning the same protein are aligned to account for overlaps, with sequence logos indicated on the top of MSA. The height of the letters corresponds to the frequency of each amino acid at that position in the sequence. Each residue is color-annotated based on hydropathy: acidic (red), basic (blue), polar uncharged (yellow), and hydrophobic nonpolar (green). "Other (ASY-specific)" depicts all the remaining peptide sequences that were ASY-specific.
(TIF)
