# Supplementary figures and images for: Antibody profiling and predictive modeling discriminate between Kaposi sarcoma and asymptomatic KSHV infection
Source: PLoS Pathog. 2024 Feb 21;20(2):e1012023. doi: 10.1371/journal.ppat.1012023 (PMC10911871; doi:10.1371/journal.ppat.1012023)

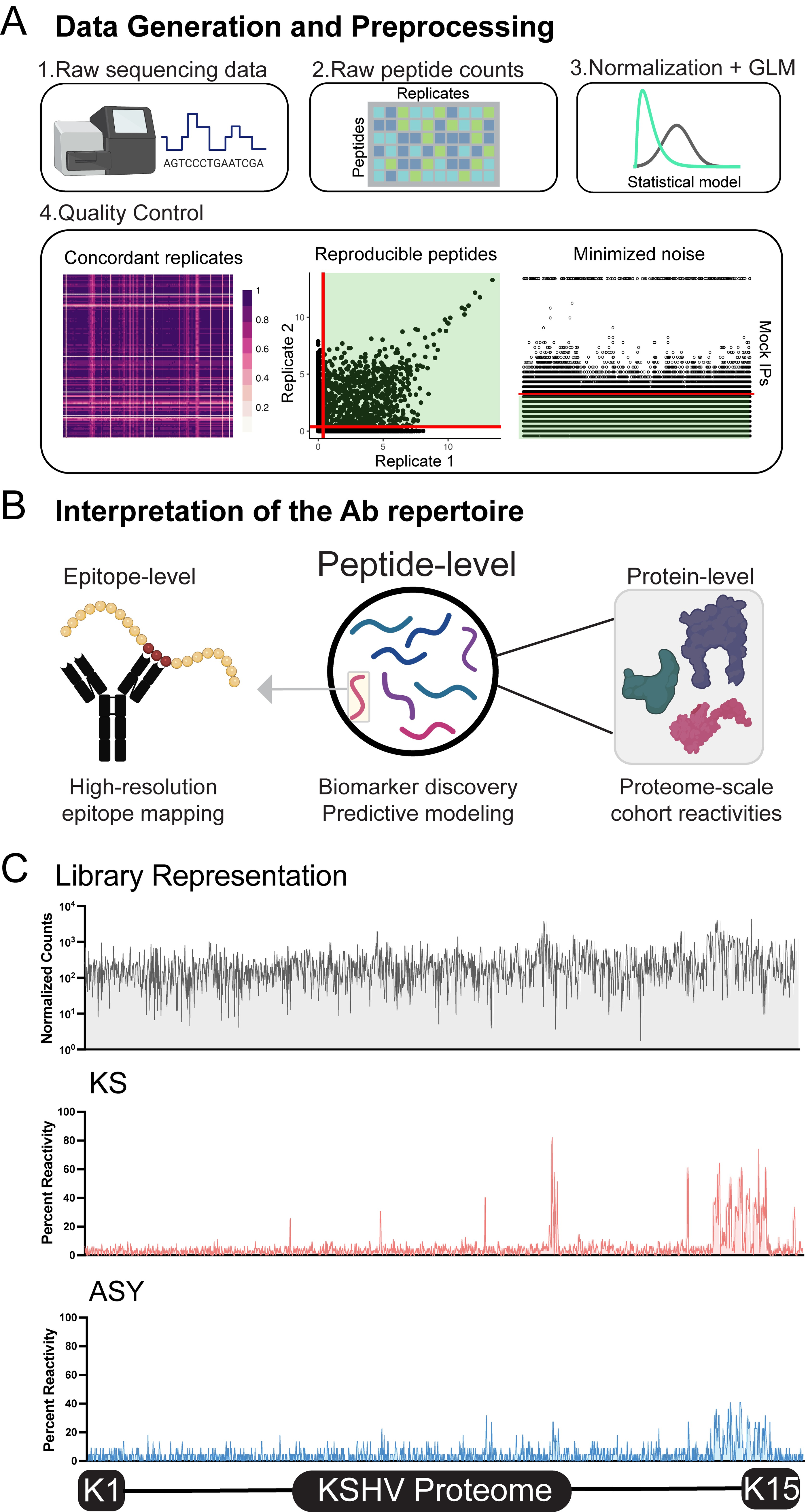

Supplement: S1 Fig — (A) The raw sequencing data was aligned to the reference oligonucleotides, and the mapped reads were counted to obtain a matrix of sample replicates by peptides. Further, raw peptide counts were normalized, and Gamma-Poisson fitted. After residual p-values were estimated, the quality of the peptides and samples was assessed. Peptides were excluded if high binding was detected in the mock IPs. Samples were excluded if the replicate correlation coefficient was less than 0.7. Created with BioRender.com. (B) The pre-processed peptide-level data was further analyzed at the protein- and epitope-levels. (C) The distribution of the normalized counts for each KSHV peptide in the library (black) and the percent reactivity against each peptide for KS (pink) and ASY (blue) is sorted by KSHV genomic order. Abbreviations: Generalized linear model (GLM), Kaposi Sarcoma (KS), asymptomatic (ASY). (TIF) [file ppat.1012023.s002.tif]

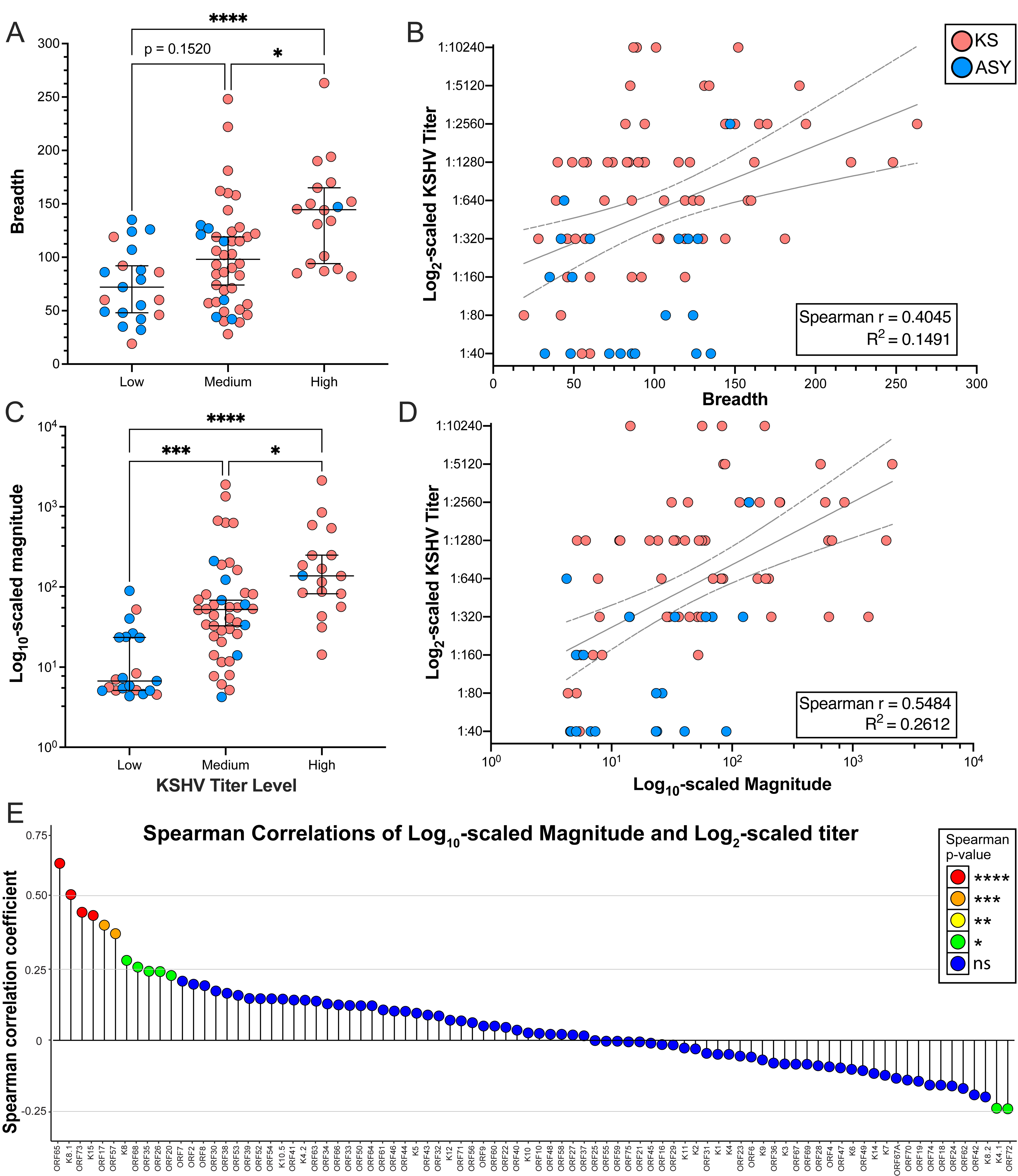

Supplement: S2 Fig — (A) The number of reactive peptides from KSHV proteins (Breadth) and (C) the frequency with which those peptides were targeted (Magnitude) were compared between individuals with low (1:40, 1:80, 1:160), medium (1:320, 1:640, 1:1280), and high (1:2560, 1:5120, 1:10240) titers. Significant comparisons were determined using Kruskal-Wallis with Dunn’s multiple comparisons post hoc test. Each data point represents an individual, and medians with interquartile ranges (IQR) are shown. The Spearman correlation and linear regression of KSHV (B) breadth and (D) magnitude with titer where the solid lines represent the goodness-of-fit and the dashed lines represent the 95% confidence intervals. (E) The average magnitude for each KSHV protein was tested for associations with titer (Spearman). The y-axis represents the correlation coefficient while the color of the point represents the statistical significance. Abbreviations: Kaposi Sarcoma (KS), asymptomatic (ASY), no statistical significance (ns), ****p<0.0001, ***p<0.001, **p<0.01, *p<0.05. (TIF) [file ppat.1012023.s003.tif]

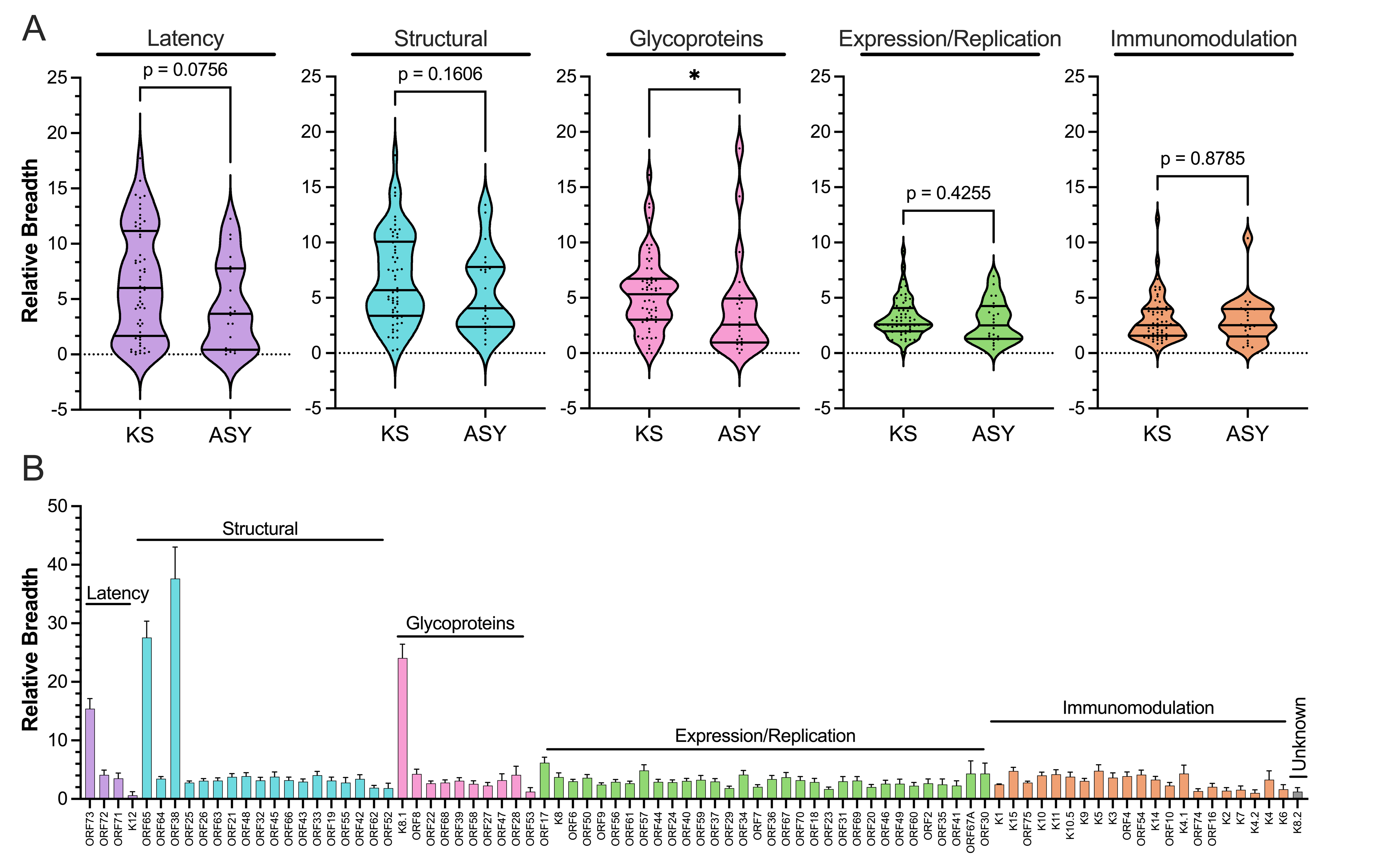

Supplement: S3 Fig — Each KSHV protein was categorized into a functional group, and the breadth of the antibody response to each functional group as well as the individual protein was compared. Significant comparisons were determined using the Friedman test with Dunn’s multiple comparisons post hoc tests in (A). The functional groups were compared between the ASY and KS cohorts. In the volcano plots, each data point represents an individual, the middle line represents the median, and the outer lines represent the quartiles. (B) Means with standard errors of the mean (SEM) are shown. Kaposi Sarcoma (KS), asymptomatic (ASY), ****p<0.0001, ***p<0.001, **p<0.01, *p<0.05. (TIF) [file ppat.1012023.s004.tif]

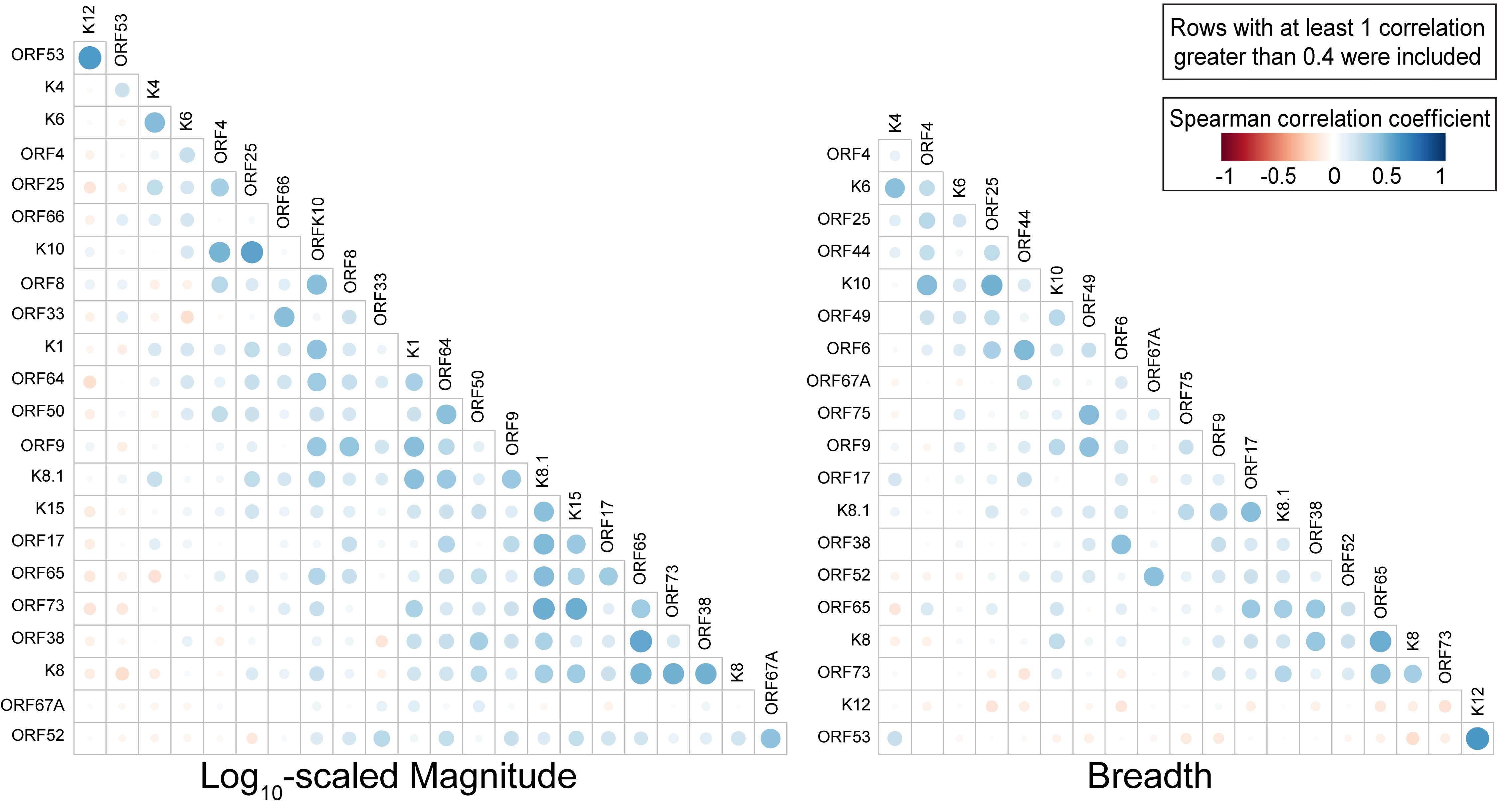

Supplement: S4 Fig — The Spearman correlation of log10-scaled magnitude and breadth for each KSHV protein with the other KSHV proteins. The proteins that only had weak to no correlation with other KSHV proteins (rs < 0.4) are not shown. Abbreviations: open reading frame (ORF). (TIF) [file ppat.1012023.s005.tif]

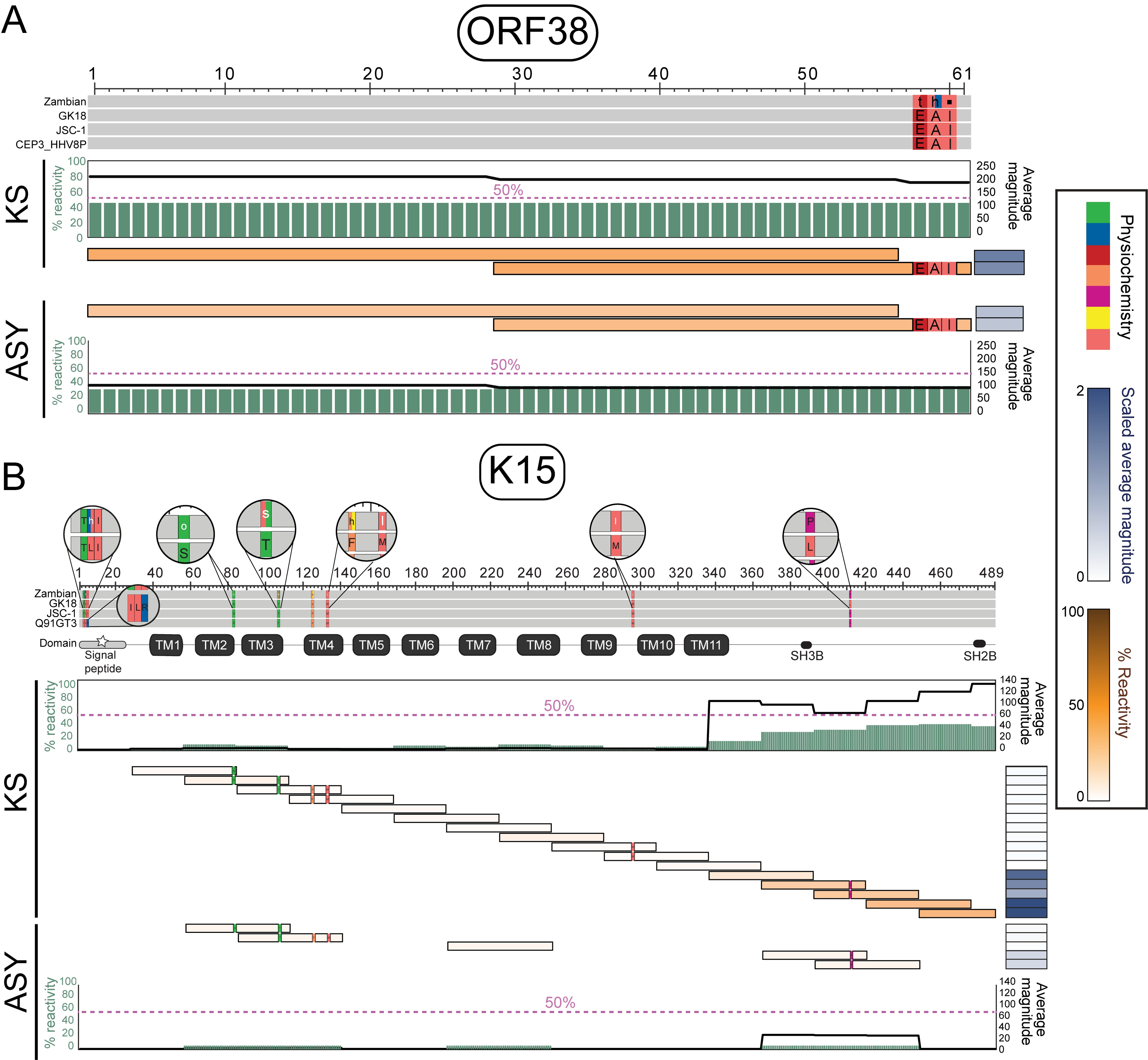

Supplement: S5 Fig — For (A) ORF38 and (B) K15, the known domains and motifs are annotated across the proteins, followed by the multiple sequence alignment (MSA) of reference (GK18, JSC-1) and input library (CEP3_HHV8P, Q91GT3) sequences. Gray represents 100% conservation among the sequences, while mismatched residues are colored by their physiochemical properties as defined by the Zappo color scheme (green: hydrophilic, salmon: aliphatic/aromatic, orange: aromatic, fuchsia: conformationally special, yellow: cysteine only, red: negatively charged, blue: positively charged). Further, each peptide targeted by at least one individual was also aligned and colored by the percent reactivity (brown). To the right, the scaled average magnitudes (blue) are shown as a heatmap. Finally, the percent reactivity (sage) and average magnitude (black lines) of the amino acid-level responses are shown for KS and ASY. Abbreviations: Kaposi Sarcoma (KS), asymptomatic (ASY), transmembrane (TM), Src homology 2/3 binding sites (SH2/3B), s: small (A/C/D/G/N/P/S/T/V), h: hydrophobic (A/C/F/G/H/I/K/L/M/R/T/V/W/Y), o: alcohol (S/T), t: turn-like (A/C/D/E/G/H/K/N/Q/R/S/T), ∎: resulted in a gap in 5/16 African samples. (TIF) [file ppat.1012023.s006.tif]

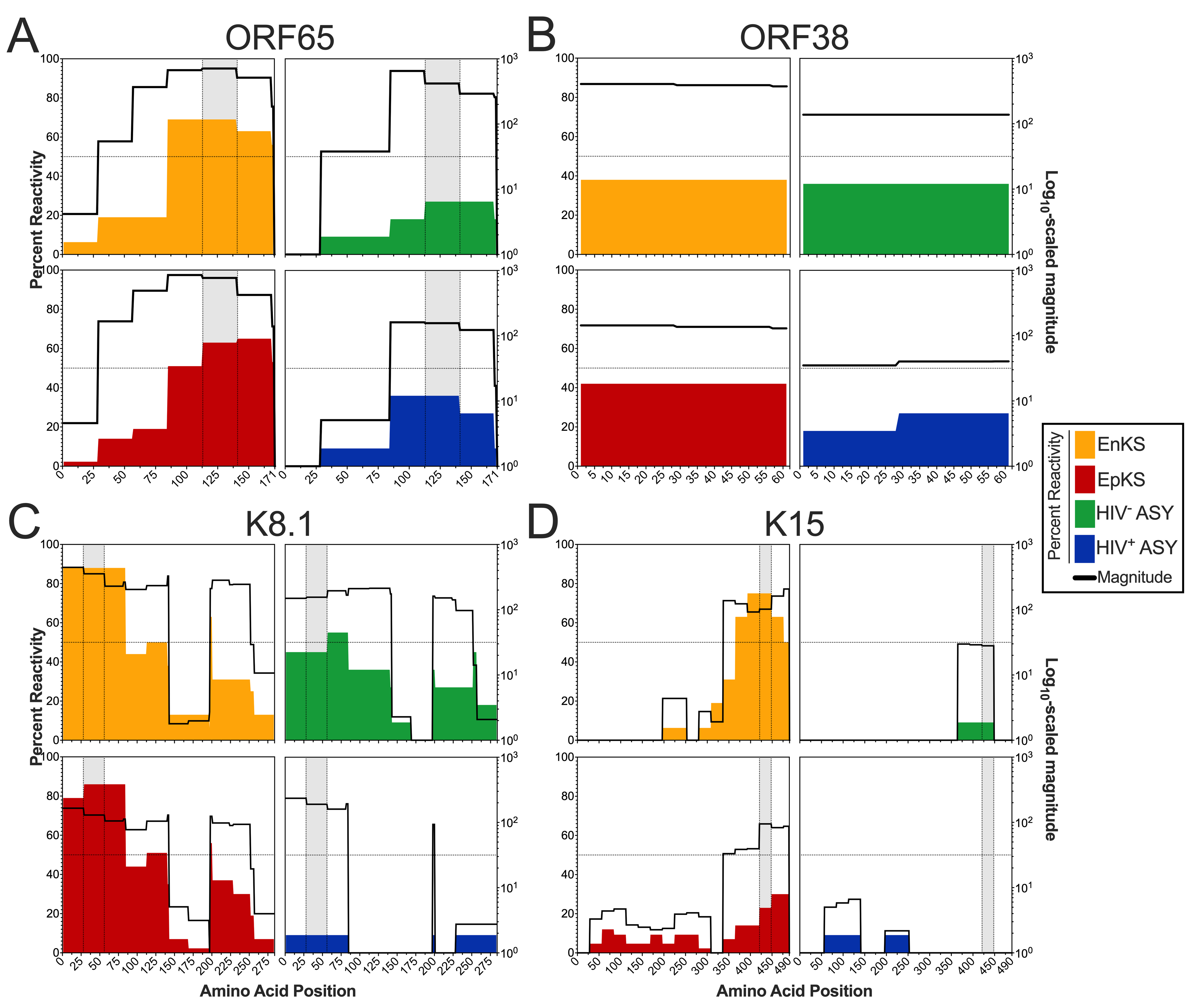

Supplement: S6 Fig — The colored areas represent the percentage of patients that were reactive to at least one peptide containing that residue (percent reactivity), while the black lines represent the log10-scaled average magnitude [log(-log(p))] at that residue for EpKS, EnKS, HIV+ ASY, and HIV- ASY in (A) ORF65, (B) ORF38, (C) K8.1, and (D) K15. The gray boxes represent the areas of the highlighted epitopes from S4 and S5 Figs. Abbreviations: Kaposi Sarcoma (KS), asymptomatic (ASY), human immunodeficiency virus 1 (HIV), endemic KS (EnKS), epidemic KS (EpKS). (TIF) [file ppat.1012023.s007.tif]

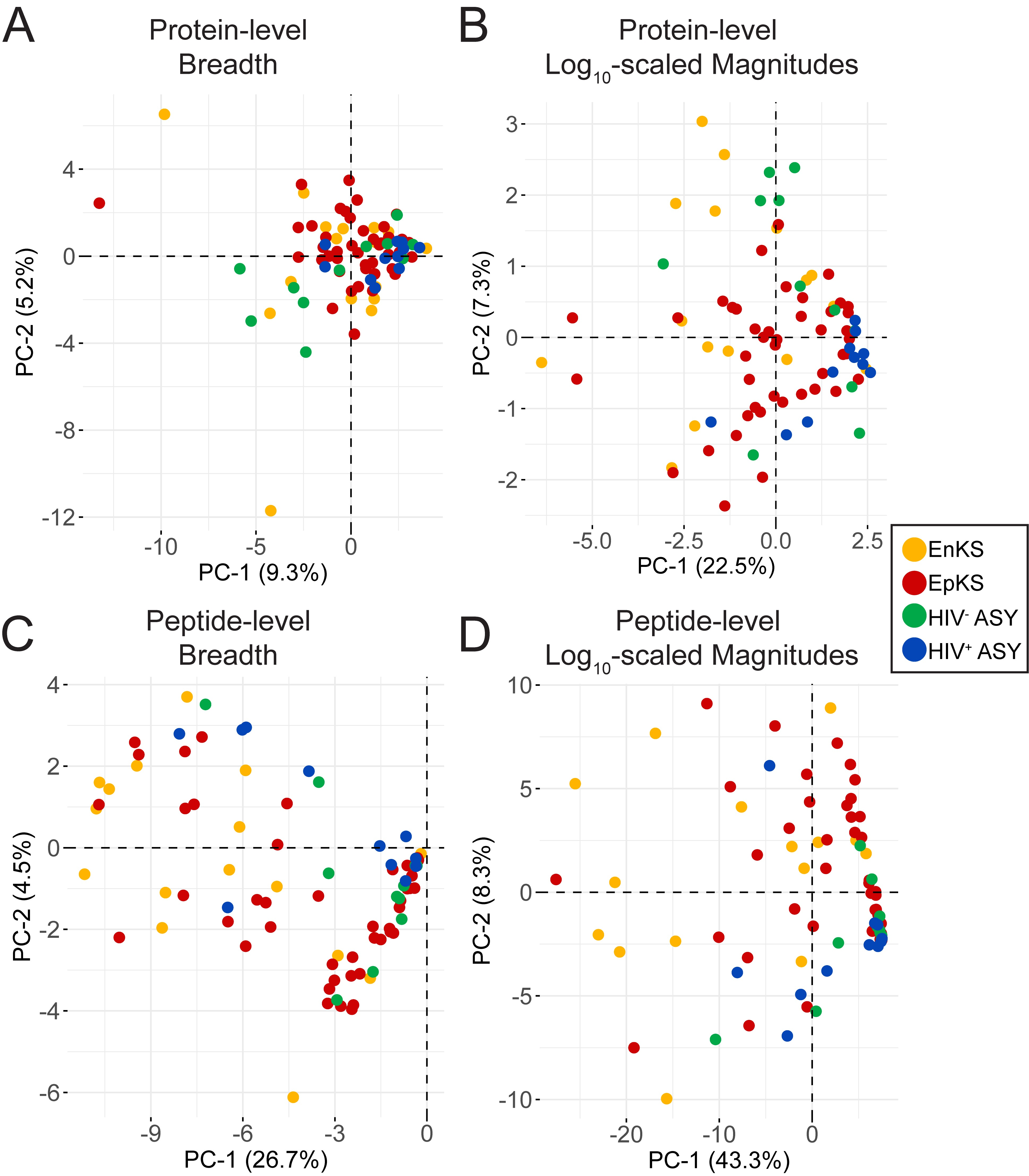

Supplement: S7 Fig — 2D principal component analysis (PCA) projections are visualized using the data comprising (A) breadth per KSHV protein, (B) log10-scaled average magnitude per KSHV protein, (C) reactive KSHV peptides, and (D) log10-scaled magnitude for each of the reactive KSHV peptides. In the PCA projections, each data point represents an individual. Abbreviations: Kaposi Sarcoma (KS), asymptomatic (ASY), human immunodeficiency virus 1 (HIV), endemic KS (EnKS), epidemic KS (EpKS), principal component (PC). (TIF) [file ppat.1012023.s008.tif]

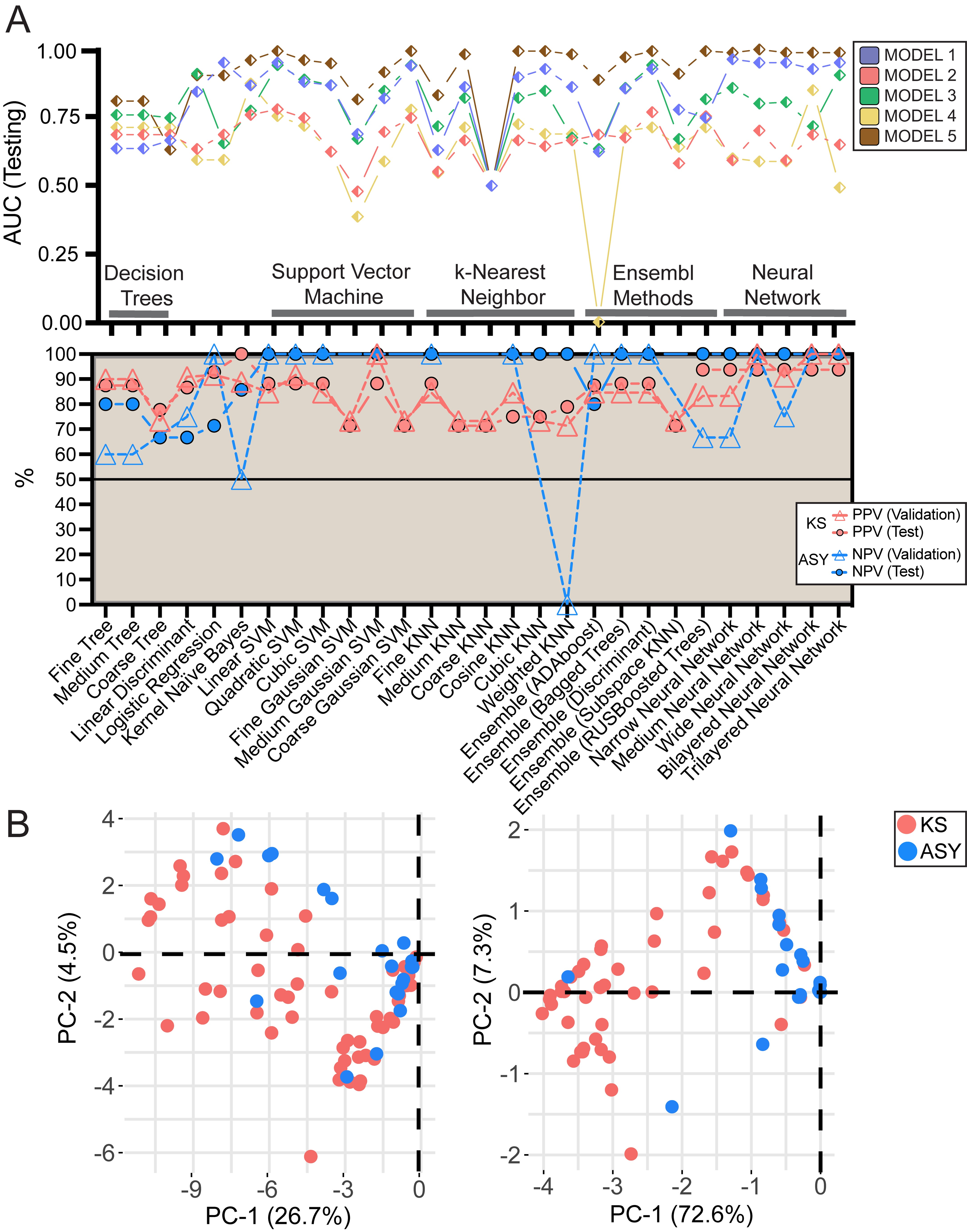

Supplement: S8 Fig — (A) Areas under the curve of each tested model are displayed across each classifier for assessing consensus performance between models. The bottom panel shows the positive and negative predictive values using validation and test sets per classifier in the top-performing model (i.e., Model 5). (B) 2D PCA projections using all 1,988 KSHV peptides as the feature set (left), compared with the 2D PCA projections using only the 25 discriminative features from the top-performing model–Model 5 (right). Abbreviations: Kaposi Sarcoma (KS), asymptomatic (ASY), area under the curve (AUC), positive predictive value (PPV), negative predictive value (NPV), support vector machine (SVM), k-nearest neighbor (KNN), principal component analysis (PCA). (TIF) [file ppat.1012023.s009.tif]

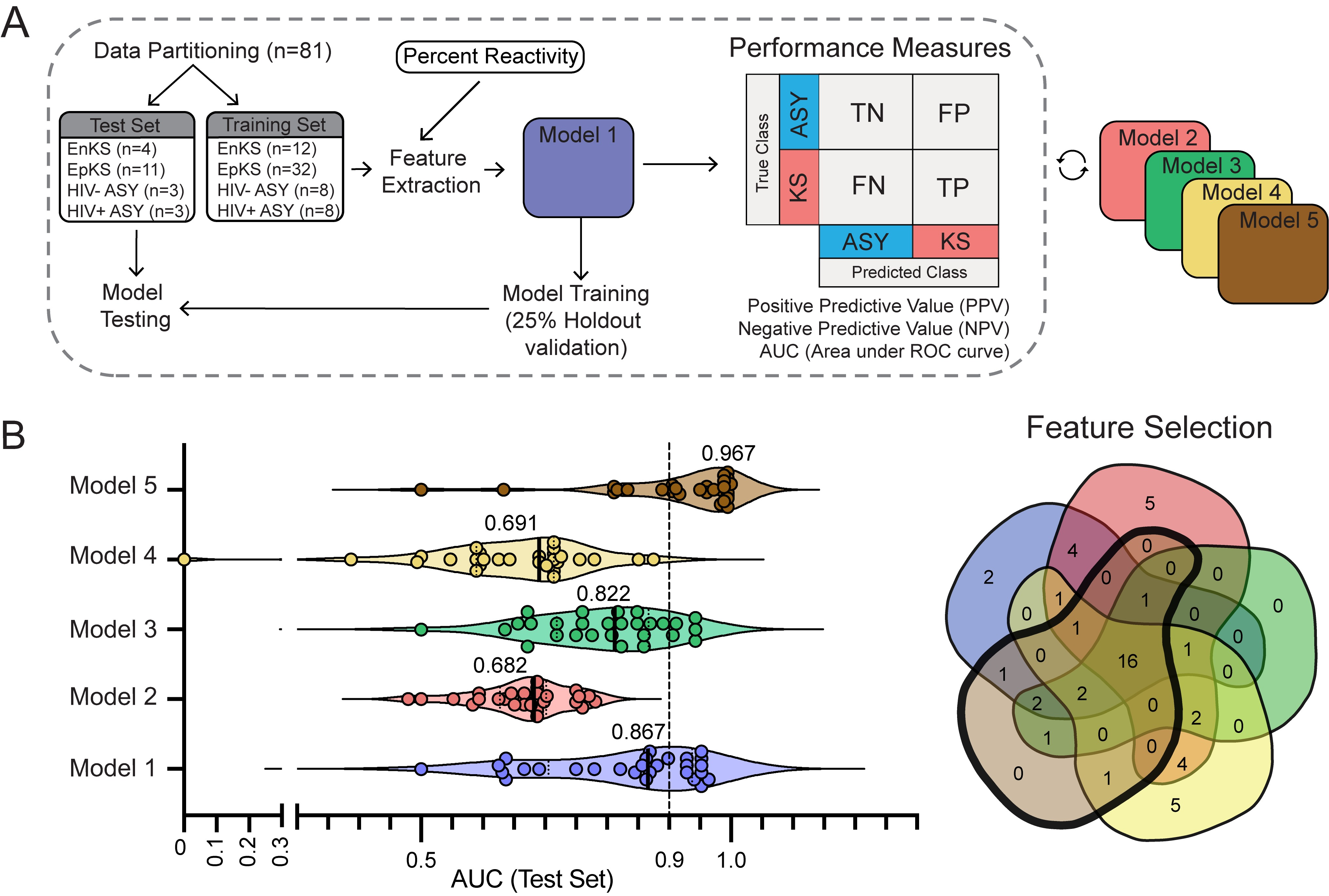

Supplement: S9 Fig — (A) The samples were divided into training (75%) and test sets (25%), preserving sub-class balance within KS and ASY. Five such datasets were generated, using combinations of samples without replacement. Training sets of each dataset were used for unbiased feature extraction. Classification models were built using those extracted features and tested on each partitioned test set. Holdout validation using 25% of the training set was applied to each training set to assess validation-test tradeoffs. The performance of each model was evaluated using positive and negative predictive values (PPV and NPV, respectively), and area under the receiver operating characteristics (ROC) curve (AUC). (B) AUCs for each classifier performance within a model were visualized using violin plots, where each point represents the AUC of a classifier and the black line and text represent the median AUC for each model (left). Number of unique or shared peptides in each model are indicated, where Model 5’s features were selected as the top discriminative peptides. Abbreviations: Kaposi Sarcoma (KS), asymptomatic (ASY), human immunodeficiency virus 1 (HIV), endemic KS (EnKS), epidemic KS (EpKS), true negative (TN), true positive (TP), false positive (FP), false negative (FN). (TIF) [file ppat.1012023.s010.tif]

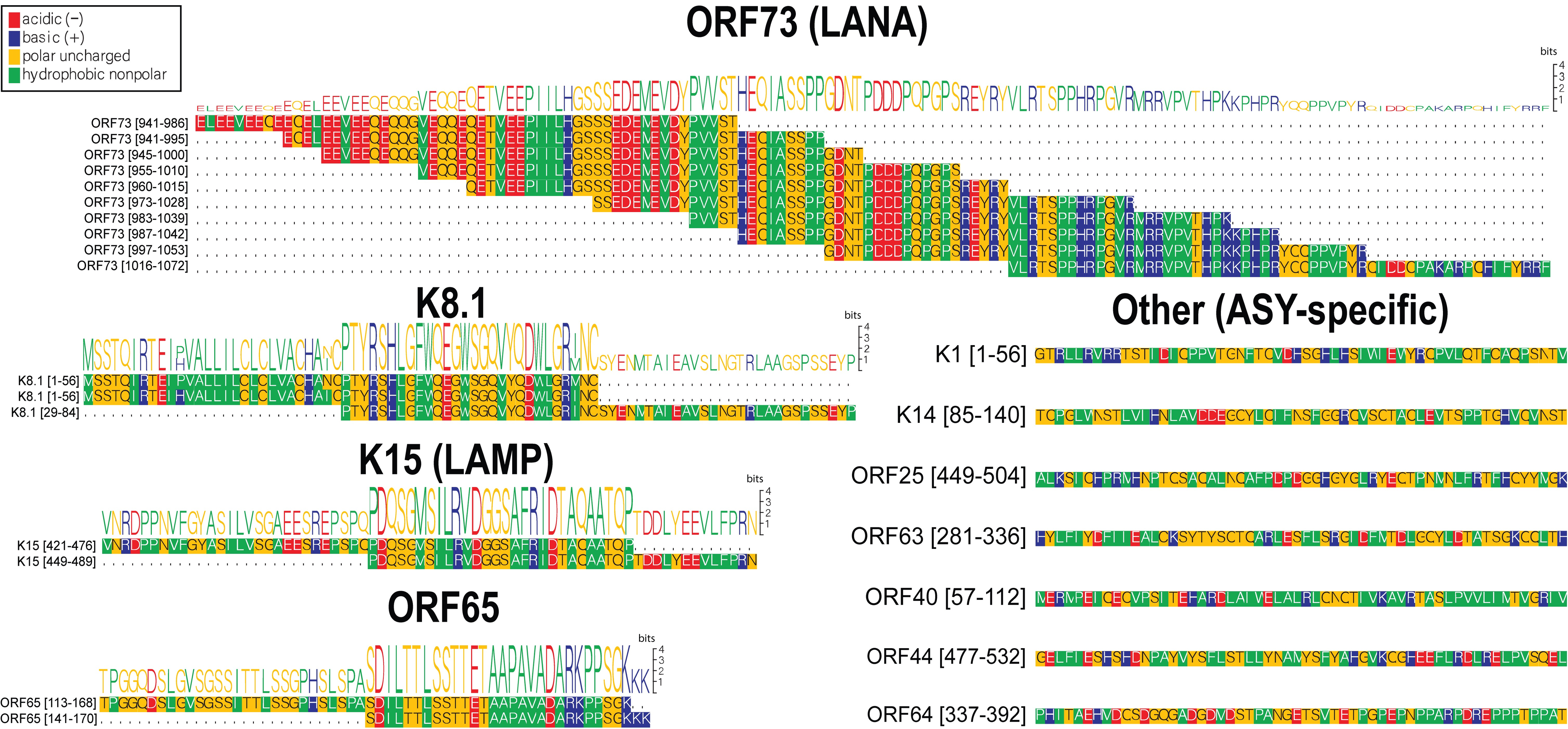

Supplement: S10 Fig — Multiple peptides spanning the same protein are aligned to account for overlaps, with sequence logos indicated on the top of MSA. The height of the letters corresponds to the frequency of each amino acid at that position in the sequence. Each residue is color-annotated based on hydropathy: acidic (red), basic (blue), polar uncharged (yellow), and hydrophobic nonpolar (green). "Other (ASY-specific)" depicts all the remaining peptide sequences that were ASY-specific. (TIF) [file ppat.1012023.s011.tif]
